# Eco-Nanozymology: A Catalytic Paradigm Integrating Energy, Environment, and Ecology

**DOI:** 10.1007/s40820-026-02269-7

**Published:** 2026-06-26

**Authors:** Limin Shang, Ziqi Zhang, Hongyu Lin, Zichang Wang, Dehong Chen, Zhiling Zhu

**Affiliations:** College of Materials Science and Engineering, Qingdao University of Science and Technology, 53 Zhengzhou Road, Qingdao, 266042 Shandong People’s Republic of China

**Keywords:** Eco-nanozyme, Ecology, Energy, Environment, Sustainability

## Abstract

Eco-nanozymology, as an emerging interdisciplinary field, integrates nanotechnology with principles of enzymology to offer innovative strategies for energy conversion and environmental remediation. In contrast with conventional enzymology, eco-nanozymology enhances catalytic efficiency through the precise regulation of interfacial microstructures, electronic distributions, active site configurations, multienzyme cascade catalysis, and functionalized carrier engineering. By harnessing and amplifying natural ecological processes, eco-nanozymology enables effective modulation of energy flow and material cycling within ecosystems. This review focuses on recent advances in the application of eco-nanozymology across energy-related domains, including nitrogen fixation, carbon fixation, methane oxidation, hydrogen production, and energy conversion devices. It further provides an in-depth analysis of the recent progress in the efficient degradation of environmental pollutants and resource-oriented valorization of low-value biomass, such as lignin, agricultural residues, livestock manure, and microplastics. These studies highlight the pivotal role of eco-nanozymology in enhancing the efficiency of environmental management, accelerating the development of green energy technologies, and advancing carbon neutrality goals. The continued development of eco-nanozymology is expected to open new horizons for the deepening and expansion of nanozyme applications, offering green, environmentally friendly, and sustainable technological pathways to address global energy and environmental challenges.

## Highlights

- Eco-nanozymology is proposed as an ecosystem-oriented framework integrating cross-scale energy and matter cycling.
- Ecological nanozymes demonstrate significant advances in energy conversion and environmental remediation.
- Eco-nanozymes enable improved remediation efficiency, green energy technologies, and carbon neutrality.

## Graphical abstract

**Figure:**
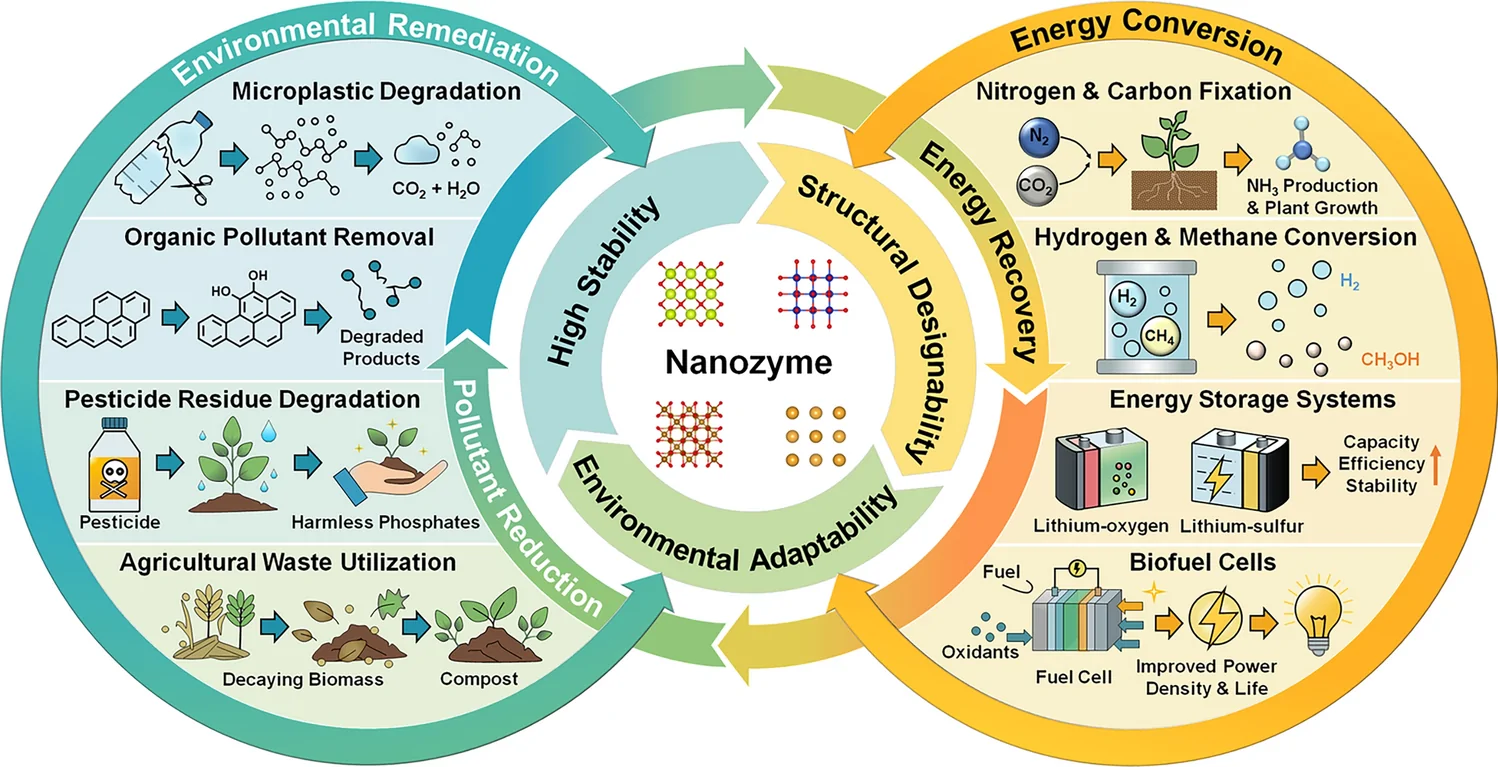

## Introduction

**Fig. 1:**
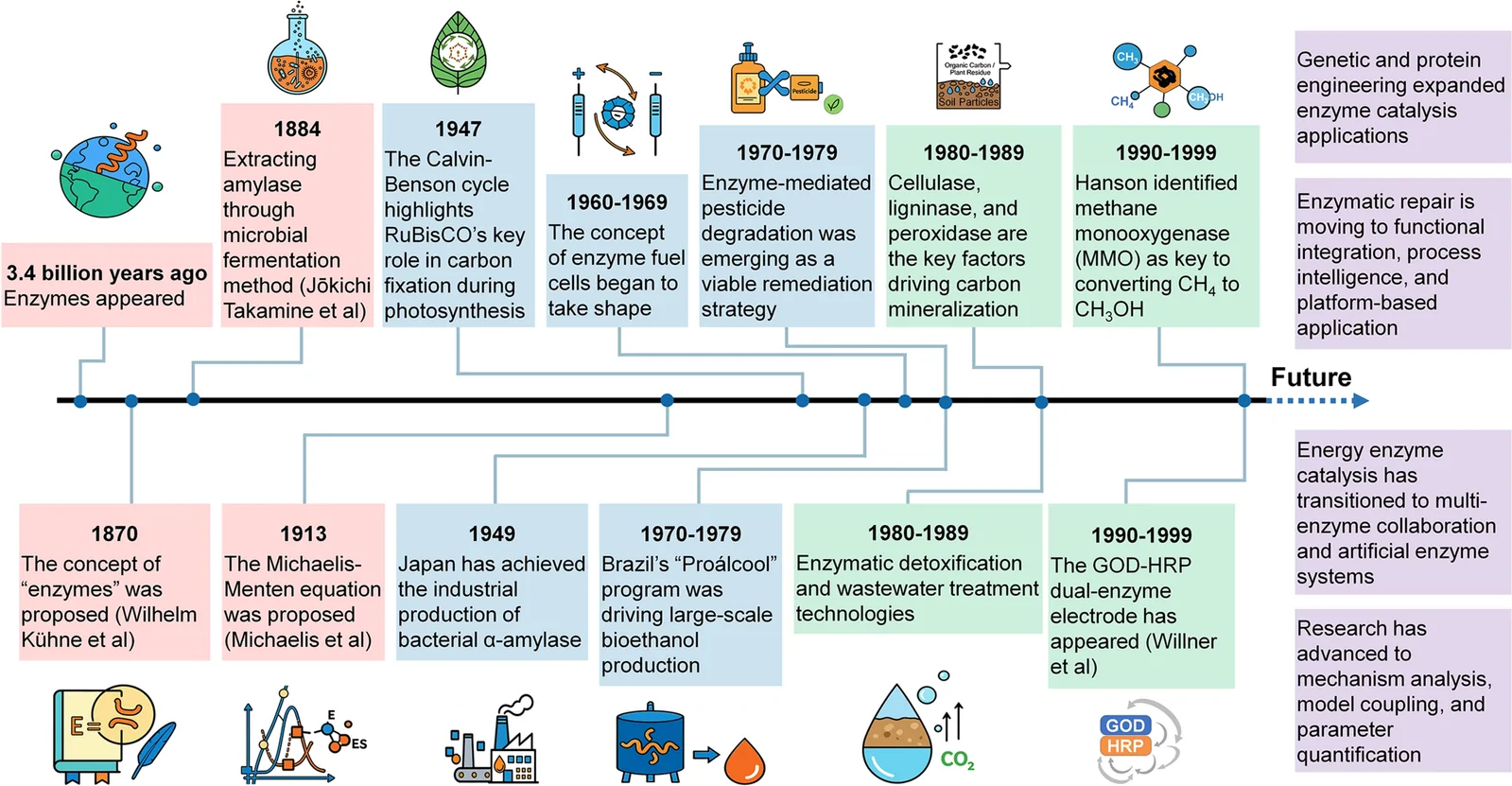
Historical trajectory of enzyme development in energy conversion and environmental remediation

Enzymes emerged on Earth approximately 3.4 billion years ago [1], nearly coinciding with the origin of life, and have since served as molecular engines driving Earth’s energy flow and material cycling [2]. However, the systematic understanding and practical utilization of enzymes by humans span a history of only slightly more than a century (Fig. 1). In the late nineteenth century, the German physiologist Wilhelm Kühne introduced the concept of “enzymes.” In 1884, the Japanese scientist Jokichi Takamine isolated amylase through microbial fermentation, pioneering the industrialization of enzyme preparations. In 1913, Michaelis and Menten proposed the Michaelis–Menten equation, establishing the theoretical foundation of enzyme kinetics. In 1949, the industrial-scale production of microbial α-amylase was achieved in Japan, marking the formal advent of the enzyme industry. By the late twentieth century, advances in genetic and protein engineering substantially expanded the scope and application of enzymatic catalysis [3].

In the field of energy conversion, enzymes serve as key biocatalytic units for constructing green energy conversion systems, demonstrating remarkable potential in biofuels and bioelectrochemical energy technologies. The concept of enzymatic fuel cells first emerged in the 1960s [4]. In 1975, Brazil’s “Proálcool” program advanced the large-scale production of bioethanol [5]. In the 1990s, lipases catalyzed the transesterification of fats and oils [6], laying the foundation for biodiesel production. In 1998, Willner and colleagues constructed a glucose oxidase-horseradish peroxidase (GOD-HRP) dual-enzyme electrode [7], enabling biocatalytic electricity generation and initiating research on enzymatic fuel cells. Subsequently, multienzyme cascade systems and biomimetic metalloenzyme systems were developed in succession [8, 9]. By the 2010s, enzymatic catalysis had transitioned from single enzymes to multienzyme cascade systems and from natural enzymes to artificial enzyme systems, with continuous improvements in energy conversion efficiency [10, 11].

In the field of environmental remediation, owing to the high selectivity, low energy consumption, and environmental compatibility, enzymes have been extensively employed in pollutant degradation, bioremediation, and the treatment of organic wastes, thereby constituting an essential biological tool for achieving green and sustainable environmental management. Since the 1970s, enzyme-mediated pesticide degradation has gradually emerged as a viable remediation strategy [12]. In the 1980s, enzyme-based detoxification approaches and wastewater treatment technologies emerged, exemplified by peroxidase (POD)-mediated degradation of phenolic pollutants. In the 1990s, laccases and lignin peroxidases were engineered and scaled up for the first time, enabling applications in hydrocarbon degradation and industrial wastewater treatment [13]. In the twenty-first century, enzymatic remediation has progressively evolved from experimental exploration toward functional integration, process intelligence, and platform-based applications. Beyond their role in pollutant removal, enzymes also play a fundamental role in global carbon cycling and climate regulation. For example, the elucidation of the Calvin–Benson cycle in 1947 revealed the central role of RuBisCO in photosynthetic carbon fixation [14]. In the 1980s, cellulases, ligninases, and peroxidases were demonstrated to be key drivers of carbon mineralization [15]. In the 1990s, Hanson discovered that methane monooxygenase (MMO) mediates the conversion of methane to methanol [16], highlighting the critical role of biological methane oxidation in maintaining atmospheric methane homeostasis. Entering the twenty-first century, research shifted from qualitative observations toward mechanistic elucidation, model integration, and quantitative parameterization. Recent evidence indicates that global warming can enhance the activity of oxidoreductases, whereas the responses of hydrolases to temperature increases exhibit pronounced ecosystem specificity and are not directionally uniform [17]. These responses are primarily regulated by local environmental factors, including substrate availability, microbial community composition, soil nutrient status, and pH. Furthermore, enzyme kinetic parameters have been incorporated into Earth system models, establishing a microbe–enzyme explicit carbon cycling framework that enables process-based simulations of temperature sensitivity, substrate limitation, and carbon use efficiency [18, 19].

Despite their remarkable potential in energy conversion and environmental remediation, the practical application of enzymes remains constrained by limitations such as insufficient stability, narrow substrate specificity, limited operational lifespan, and relatively high cost. To translate their intrinsic catalytic advantages into effective system-level performance, it is critical to consider how environmental factors and system design affect enzyme activity. In complex environments characterized by high salinity, elevated temperatures, or strong oxidative conditions, natural enzymes are prone to denaturation and inactivation. Moreover, in energy systems, long electron transport pathways and high internal resistance result in enzyme-assisted platforms exhibiting energy conversion efficiencies far below theoretical limits.

With the convergence of nanotechnology, materials science, and life sciences, nanozymes, as nanomaterials with enzyme-like catalytic properties [20, 21], offer a novel strategy to overcome the intrinsic limitations of natural enzymes. Their high stability, structural tunability, and environmental adaptability make them a versatile platform for substituting natural enzymes [3, 22–24]. In energy conversion, nanozymes can significantly reduce electrochemical impedance and enhance energy conversion efficiency through optimized electron transport [25], multienzyme cascade system [23], and enzyme–electrode interface engineering [26], providing a new paradigm to overcome bottlenecks in bioenergy technologies. In environmental remediation, nanozymes can achieve resistance to inactivation through structural biomimicry [27] and interfacial engineering [26], while inorganic or hybrid frameworks withstand extreme conditions. Porous architectures and tunable surfaces enable substrate enrichment and microenvironmental regulation [28], and magnetic or carrier immobilization facilitates recyclability and sustainable catalysis.

## Concept and Development of Eco-Nanozymology

### Conceptual Proposal and Definition

Nanozymes are an emerging research field arising from the intersection of nanotechnology and enzymology, with significant potential in energy conversion [25, 29], environmental remediation [30, 31], and biomedical applications [32–34]. On this basis, the concept of “eco-nanozymology” is introduced to systematically define the catalytic behaviors, functional evolution, and environmental adaptability of nanozymes at the ecosystem level. Eco-nanozymology aims to achieve the amplification of catalytic efficiency within ecological processes through the precise modulation of interfacial microstructures, electronic distributions, active site configurations, multienzyme cascade catalysis, and functionalized carrier engineering, thereby enabling artificial regulation of energy flow and material cycling in ecosystems. The establishment of this concept not only broadens the application scope of nanozyme research but also provides a new theoretical framework for molecular-level regulation of ecological processes. Importantly, defining “eco-nanozymology” as an independent conceptual framework is necessary, as it enables a systematic distinction between ecosystem-level catalytic regulation and traditional nanozyme research focused on material design or single-reaction systems, thereby clarifying its scientific boundary and theoretical positioning and providing support for the development of its theoretical framework.

### Fundamental Distinctions Between Eco-Nanozymology and Related Concepts

To clarify the conceptual boundary and research positioning of eco-nanozymology, this section systematically distinguishes related catalytic systems in terms of research scale and functional focus.

#### Catalysts for Energy Conversion and Environmental Remediation

Catalysts for energy conversion and environmental remediation typically refer to functional materials that promote specific chemical transformations by tuning the structure of active sites, surface electronic properties, and interfacial reaction processes [35–37]. By constructing efficient catalytic centers and adjusting electronic structure and substrate adsorption, these materials can lower reaction barriers and optimize reaction pathways, thereby achieving high efficiency and selectivity in energy conversion or pollutant degradation [38, 39]. Current research primarily correlates catalytic performance with material composition, micro- and nanostructure, and surface physicochemical properties, focusing on optimization and mechanistic understanding at the material- or reaction-specific level, while system-level environmental feedback remains largely unexplored.

#### Bioinspired Catalysis Within Ecosystems

Among various catalytic strategies, bioinspired catalysis within ecosystems is a class of techniques that combines features of chemical and biological catalysis [40, 41]. It can achieve high selectivity under mild conditions. Its core lies in designing catalysts that mimic enzyme functional units, establishing enzyme-like reaction mechanisms [42], and creating microenvironments similar to natural enzyme systems. Typical approaches include using single-atom catalysts to construct non-contact reaction pathways or employing porous materials to simulate enzyme active centers, microenvironments, and mass transport channels [43]. These strategies combine the high selectivity of biocatalysis with the industrial applicability of chemical catalysis, enabling selective hydrocarbon oxidation, pollutant degradation, and energy conversion [44]. However, this approach remains fundamentally reaction centered, with its primary focus placed on optimizing catalytic efficiency within isolated reaction environments or reactor systems. Notably, it does not explicitly incorporate system-level feedback regulation linking catalytic activity with environmental transformation networks and is therefore largely constrained to localized catalytic optimization.

#### Eco-Nanozymology

Eco-nanozymology extends bioinspired catalysis from reaction-centered systems to a process network-level framework governed by feedback-regulated environmental interactions. In contrast with bioinspired catalysis, which is primarily confined to optimizing catalytic performance within isolated reaction environments, eco-nanozymology explicitly incorporates feedback coupling between nanozyme-mediated reactions and system-level matter and energy fluxes within environmental transformation networks. Within this framework, nanozymes are conceptualized as embedded regulatory nodes in coupled environmental transformation networks rather than isolated catalytic entities. They not only mediate molecular transformations but also participate in the regulation of interconnected environmental processes through coupled catalytic and interfacial effects. Through rational design, nanozyme systems are developed beyond activity optimization, with integrated consideration of environmental compatibility and long-term system sustainability. This framework enables a transition from reaction-level optimization to feedback-governed system-level regulation of energy flow, nutrient cycling, and pollutant transformation in complex environmental systems.

### Theoretical Basis for the Design of Eco-Nanozymology

In natural ecosystems, microorganisms drive organic matter decomposition via the secretion of enzymes and regulate the biogeochemical cycling of key elements such as carbon (C), nitrogen (N), and phosphorus (P) [45, 46]. Ecological stoichiometry and eco-enzymatic stoichiometry theories indicate that microorganisms modulate enzyme secretion and activity in response to imbalances in elemental ratios, thereby influencing organic matter degradation and nutrient release [47]. Based on this framework, nanozymes can be designed to mimic natural enzyme active centers, simulating or enhancing key enzymatic reactions in ecosystems, with catalytic functions specifically tailored to address potential rate-limiting steps in material cycling. Moreover, integrating eco-enzymatic stoichiometry models and the threshold elemental ratio (TER) theory with nanozyme systems offers an experimental platform for quantitatively investigating C, N, and P cycling dynamics and validating ecological model predictions, thereby fostering a deeper integration of nanozyme design with ecological theory [48].

## Research Objectives and Guiding Principles

Eco-nanozymology, centered on bioinspired catalysis, achieves significant enhancement in catalytic efficiency by precisely regulating interfacial microstructures, electronic distributions, and active site configurations. It also emphasizes multienzyme cascades, ecological network modeling, and microenvironmental modulation to construct catalytic systems with multi-responsiveness, tunability, and evolvable characteristics. Moreover, eco-nanozymology prioritizes the synergistic compatibility between materials and their environment, promoting the coordinated operation of energy conversion and environmental remediation within an integrated energy–environment–ecology framework. This approach enables the transition from enhancing single functionalities to regulating complex ecological processes, ultimately progressing toward self-healing, system-integrated artificial catalytic platforms.

## Research Scope and Development Trends

**Fig. 2:**
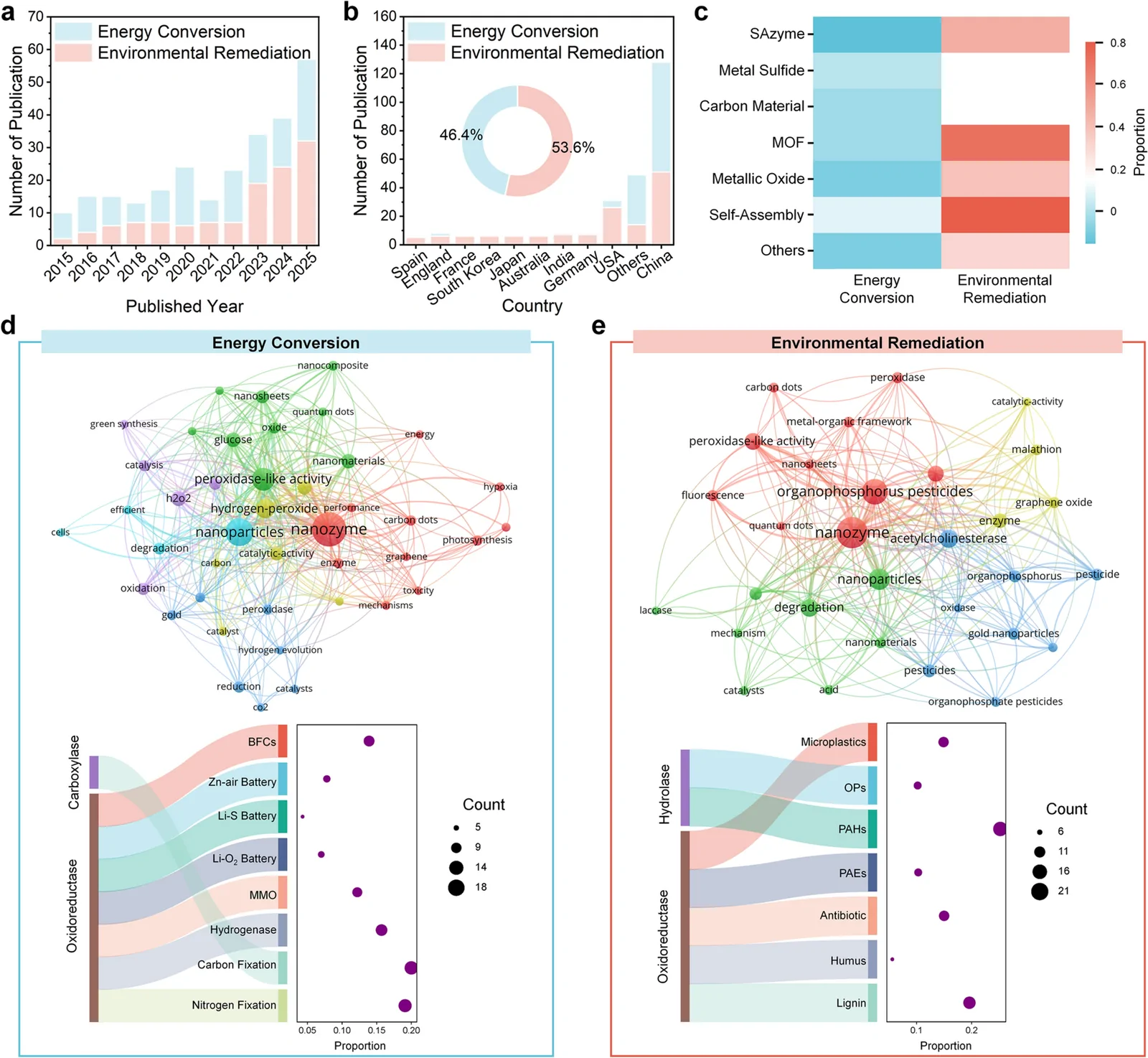
Research progress of eco-nanozymes in energy conversion and environmental remediation.** a** Publication trends of nanozyme-related studies in the fields of energy conversion and environmental remediation. **b** Number of publications on nanozymes in energy conversion and environmental remediation across different countries, along with the relative contribution of nanozymes to these two fields. **c** Proportional distribution of nanozyme material types in studies on energy conversion and environmental remediation. **d** Research domain distribution of nanozymes in energy conversion (top) and the proportional distribution of reaction types and applications in energy conversion (bottom). **e** Research domain distribution of nanozymes in environmental remediation (top) and the proportional distribution of reaction types and applications in environmental remediation (bottom)

The research scope of eco-nanozymology lies in elucidating how nanozymes exert catalytic functions in ecologically relevant processes as well as in defining the mechanisms through which they influence energy conversion, environmental remediation, and their coupling with ecosystem processes. To analyze development trends in the field, a literature survey (2015–2025) using keywords “nanozyme,” “artificial enzyme,” “energy,” and “environment” indicates a sustained increase in both publication number and citation counts (Fig. 2a). In terms of geographic distribution, China leads the research in nanozyme applications for energy conversion and environmental remediation, contributing over 49% of the published studies (Fig. 2b). Further analysis reveals that the types of nanozyme materials in energy conversion and environmental remediation have become increasingly diverse (Fig. 2c), with single-atom catalysts and self-assembled nanomaterials emerging as hotspot research directions, reflecting a shift from simple material construction to structural refinement and functional programmability. Keyword co-occurrence networks (Fig. 2d) illustrate that research on nanozymes in the energy sector centers on POD-like activity, integrating material structural regulation, catalytic mechanisms, and energy-environment applications, demonstrating a trend toward high interdisciplinarity and multifunctional synergy. Application mapping further shows that oxidoreductase-like nanozymes predominantly drive nitrogen fixation, hydrogen production, methane oxidation, and electrochemical processes, whereas carboxylase-like nanozymes play key roles in carbon fixation-related processes. In environmental remediation, keyword co-occurrence networks (Fig. 2e) highlight that nanozyme research focuses on the degradation of organophosphate pesticides, emphasizing POD-like activity, material structural regulation, and catalytic degradation mechanisms, often in synergy with natural enzymes. Application mapping further reveals functional differentiation and synergistic interactions between hydrolase- and oxidoreductase-like nanozymes, enabling nanozymes to perform targeted catalytic degradation of diverse pollutant types. On this basis, representative them material types and comparative catalytic activities in energy conversion (Table 1) and environmental remediation (Table 2) are summarized, providing a comprehensive overview of the research scope, functional characteristics, and future development trends of eco-nanozymology.

**Table 1:** Performance comparison of different types of nanozymes in the field of energy conversion

Application	Type of nanozyme	Enzymatic activity	Remark	References
Nitrogen fixation	10%Fe-MoSe_2_	Nitrogenase	NH_3_ production rate: 3.38 μg cm^−2^ h^−1^, Faraday efficiency: 30.8%	[49]
	MoO_2_/FeS_2_/GA	Nitrogenase	NH_3_ production rate: 40.18 μg cm^−2^ h^−1^, Faraday efficiency: 37.44%	[50]
	Cu NCs&SAs@CNFs	Nitrogenase	NH_3_ production rate: 31.45 μg cm^−2^ h^−1^, Faraday efficiency: 97.23%	[51]
	PMo_10_V_2_@MIL-88A	Nitrogenase	NH_3_ production rate: 50.82 μmol g^−1^ h^−1^	[52]
	CeO_x_/Mn_3_O_4_	Nitrogenase	H_2_O_2_ (*K*_*m*_: 7.212 mM, *V*_max_: 5.882 μM s^−1^)	[53]
	Ag_2_Se@PANi	Nitrogenase	NH_3_ production rate: 0.48 ± 0.05 μg mL^−1^	[54]
Carbon fixation	Cu_6_-Cotpy	Oxidoreductase	The generation rate of CO: 740.7 μmol g^−1^ h^−1^, Catalytic time: More than 188 h	[55]
Hydrogen production	NiCo_2_O_4_@Al-Sn	Hydrogenase	Hydrogen production rate: 915 L h^−1^ g^−1^	[56]
	OVs-TiO_2_/Pd	Hydrogenase, Oxidase	TMB (*K*_*m*_: 0.129 mM)	[57]
Methane conversion	Cu_1_/CN	Methanol monooxygenase	TOF: 405.3 ± 8.2 h^−1^	[58]
Li–O_2_ battery	Zn-TCPP(Fe)	Superoxide dismutase	The specific capacity after 100 cycles of 100 mA g^−1^ is 1000 mAh g^−1^	[59]
Li–S battery	β-CD@CoPC	Oxidase, Peroxidase	The specific capacity after 200 cycles of 0.2 C is 991 mAh g^−1^	[60]
	S@CoB_1_N_3_-MR/FNN	Hydrolase	The specific capacity after 60 cycles of 0.2 C is 1117.37 mAh g^−1^	[61]
	Fe-TCPP@Cu-BTC	Oxidase	The specific capacity after 150 cycles of 0.2 C is 935 mAh g^−1^	[62]
Zn–air battery	FePc-THDMA-COF-QA	Oxidase	Half-wave potential: 0.94 V (vs RHE), Power density: 211.5 mW cm^−2^	[63]
	SA-Fe-N_5_	Oxidase	Half-wave potential: 0.88 V (vs RHE), Power density: 217.8 mW cm^−2^	[64]
BFCs	FeN_5_ SAs	Oxidase	Half-wave potential: 0.67 V (vs RHE), Power density: 149.2 ± 4.0 μW cm^−2^	[65]
	Rh SANs	Glucose oxidase	Power density: 135.0 ± 3.0 μW cm^−2^	[66]
	G/CNTs	Glucose oxidase, Oxidase	Half-wave potential: 0.65 V (vs RHE), Power density: 64.2 μW cm^−2^	[67]

**Table 2:** Performance comparison of different types of nanozymes in the field of environmental remediation

Application	Type of nanozyme	Enzymatic activity	Remark	References
Lignin	Cu/GMP-MOFzyme	Laccase	Achieve a high yield of 81.7 wt% of lignin oligomers	[68]
	Fe–N/O/Cl–C SAzyme	Laccase	*K*_cat_*/K*_*m*_: 1.75 × 10^7^ M^−1^ min^−1^	[69]
	CotA + λ-MnO_2_	Laccase	The degradation rate reached 25.15%, and the proportion of aromatic products was 48%	[70]
	COHB	Laccase	TOF: 6.01 × 10^–3^ s^−1^, *K*_*m*_: 0.24 mM	[71]
Humus	FeS_2_/Pal	Glutathione peroxidase, Catalase	The total degradation rate of cellulose was 52.54%	[72]
	Ch Lac-NPs	Laccase	*K*_cat_*/K*_*m*_: 4840 mM^−1^ min^−1^	[73]
Antibiotics	50N-BFO	Peroxidase, Oxidase	The degradation rate of methylene blue reached as high as 94.27%, and the degradation rate of various antibiotics exceeded 60%	[74]
	CoNi-MOF	Laccase	After 30 min of continuous treatment, the TOC degradation efficiency of simulated antibiotic wastewater was 80.59%, 61.78%, and 61.44%	[75]
PAEs	Lac@PtCo@DMSN	Laccase	The degradation rate reached 81.83% within 72 h	[76]
PAHs	Cellulose-CD-MMT	Laccase	*K*_*m*_: 0.2876 mmol L^−1^, *V*_max_: 0.4467 μmol L^−1^ min^−1^	[77]
OPs	Mn uNF/Si	Hydrolase	Glyphosate of 67%, dichlofenthion of 45%, chlorpyrifos of 43%, dimethoate of 47%	[78]
	UiO-66 (Ce)/PVDF	Hydrolase	A high hydrolysis efficiency of 94.03% and a catalytic constant (*K*_cat_) of 4.9 × 10^–4^	[79]
	Eu@Ce/UiO-67	Hydrolase	*K*_*m*_: 0.24 mM, *V*_max_: 0.09 µm s^−1^	[80]
Microplastics	Fe_3_O_4_	Peroxidase	The degradation efficiency of microplastics is close to 100%, and it can be efficiently recycled and reused through magnetic means	[81]
	Cu SAs	Laccase	In the polystyrene degradation experiment, an ultra-high mineralization rate of over 90% was achieved. The degradation products were CO_2_, water, and short-chain carboxylic acids, with no toxic intermediates being produced	[82]
	G/Cu/GDY	Laccase	Within 20 h, the molecular weight of LDPE significantly decreased, transforming from a polymer to low molecular weight fragments	[83]

**Fig. 3:**
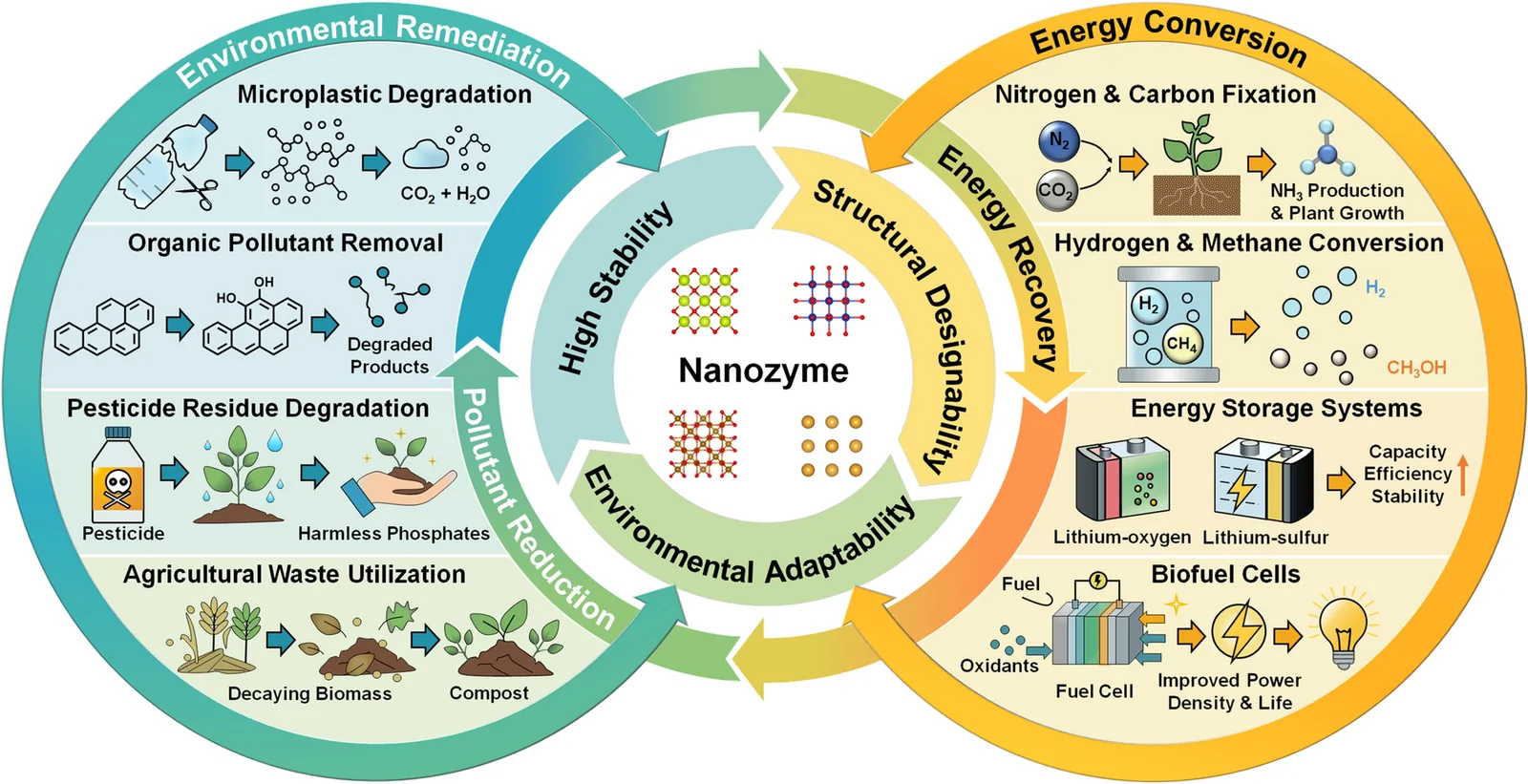
Research advances of eco-nanozyme in environmental remediation and energy conversion

In the field of energy conversion, eco-nanozymology leverages molecular design, interfacial engineering, and system integration to harness the enzyme-like catalytic advantages of nanozymes, enabling efficient and sustainable energy capture and conversion (Fig. 3). In nitrogen fixation, nanozymes mimic the proton-coupled electron transfer (PCET) mechanism of nitrogenase to activate N_2_, while utilizing oxidase (OXD)-like activity to regulate reactive oxygen species (ROS) and optimize electron transfer, thereby significantly enhancing ammonia (NH_3_) production and symbiotic nitrogen fixation efficiency. In carbon fixation, nanozymes mimic key enzymes in photosynthesis to promote CO_2_ reduction and organic carbon generation, strengthening carbon assimilation in plants and microorganisms. In hydrogen and methane oxidation, hydrogenase-like nanozymes improve the stability of hydrogen production and hydrogenation reactions, whereas MMO-like nanozymes selectively oxidize methane to methanol under mild conditions. Furthermore, in metal–air and energy storage systems, nanozymes mimic active centers such as superoxide dismutase (SOD), heme, or sulfur-oxidizing enzymes to regulate Li_2_O_2_/Li_2_S_x_ formation and decomposition, suppress side reactions, and thereby enhance the capacity, efficiency, and cycling stability of lithium–oxygen (Li–O_2_), lithium–sulfur (Li–S), and zinc–air (Zn–air) batteries. In biofuel cells (BFCs), OXD- and reductase-like nanozymes accelerate fuel oxidation and electron transfer, markedly improving power density and operational lifetime.

In the field of environmental remediation, eco-nanozymology centers on enzyme-like catalysis to achieve efficient degradation of environmental pollutants and resource-oriented valorization of low-value biomass (Fig. 3). For microplastics, hydrolase- and POD-like nanozymes can cleave C–C bonds and associated functional group linkages within polymers, converting microplastics into CO_2_, water, and low molecular weight organic acids. For organic pollutants, lipase- and phosphatase-like nanozymes catalyze the cleavage of P-O or aromatic C–C bonds, enabling efficient degradation of recalcitrant contaminants such as phthalates (PAEs), polycyclic aromatic hydrocarbons (PAHs), and antibiotics. For organophosphorus pesticides (OPs) residues, phosphatase-like nanozymes accelerate P-O bond hydrolysis, transforming toxic intermediates into harmless phosphates and providing sustainable solutions for green agriculture. In the valorization of agricultural wastes, composting, and greenhouse gas mitigation, laccase (LAC)-and POD-like nanozymes facilitate the degradation of lignocellulosic biomass and humus formation.

## Applications of Eco-Nanozymology

In this section, representative applications are discussed from an eco-nanozymology perspective. These examples are not intended to redefine existing systems, but to reinterpret their functions within a cross-scale framework linking nanozyme-catalyzed processes to system-level environmental and energy behaviors, where nanozymes act as functional units bridging molecular catalysis and macroscopic process responses.

### Nanozyme-Mediated Ecological Material Cycling

Material cycling in ecosystems involves the dynamic flow, transformation, and regeneration of essential elements, including carbon, nitrogen, and oxygen, between biotic communities and the abiotic environment. Recent studies indicate that nanozymes can emulate the active sites of nitrogenase under ambient conditions to facilitate N_2_ activation and reduction [84], while modulating plant carbon metabolic pathways to optimize photosynthetic efficiency and stability [85]. Additionally, their functional mimicry of hydrogenases or methane monooxygenases enables H_2_ production, methane oxidation, and organic matter conversion, thereby achieving effective coupling between energy turnover and biogeochemical cycling. Owing to their remarkable environmental stability, tunable architectures, and synergistic multi-enzyme activities, nanozymes exhibit substantial potential for artificial regulation of material cycles and for enhancing ecosystem functional resilience, providing innovative avenues for sustainable resource management and environmental governance.

#### Nanozyme-Mediated Artificial Nitrogen Fixation and Symbiotic Nitrogen Fixation

The nitrogen cycle is the most energy-intensive and catalytically complex biogeochemical cycle in ecosystems, with nitrogen fixation representing the key rate- determining step. Nanozymes, by biomimetically constructing nitrogenase active centers and electron transfer pathways, can activate and reduce N_2_ under mild conditions, serving as a crucial technological bridge between natural and artificial nitrogen fixation. Current research primarily advances along two directions: artificial nitrogen fixation and symbiotic nitrogen fixation.

**Fig. 4:**
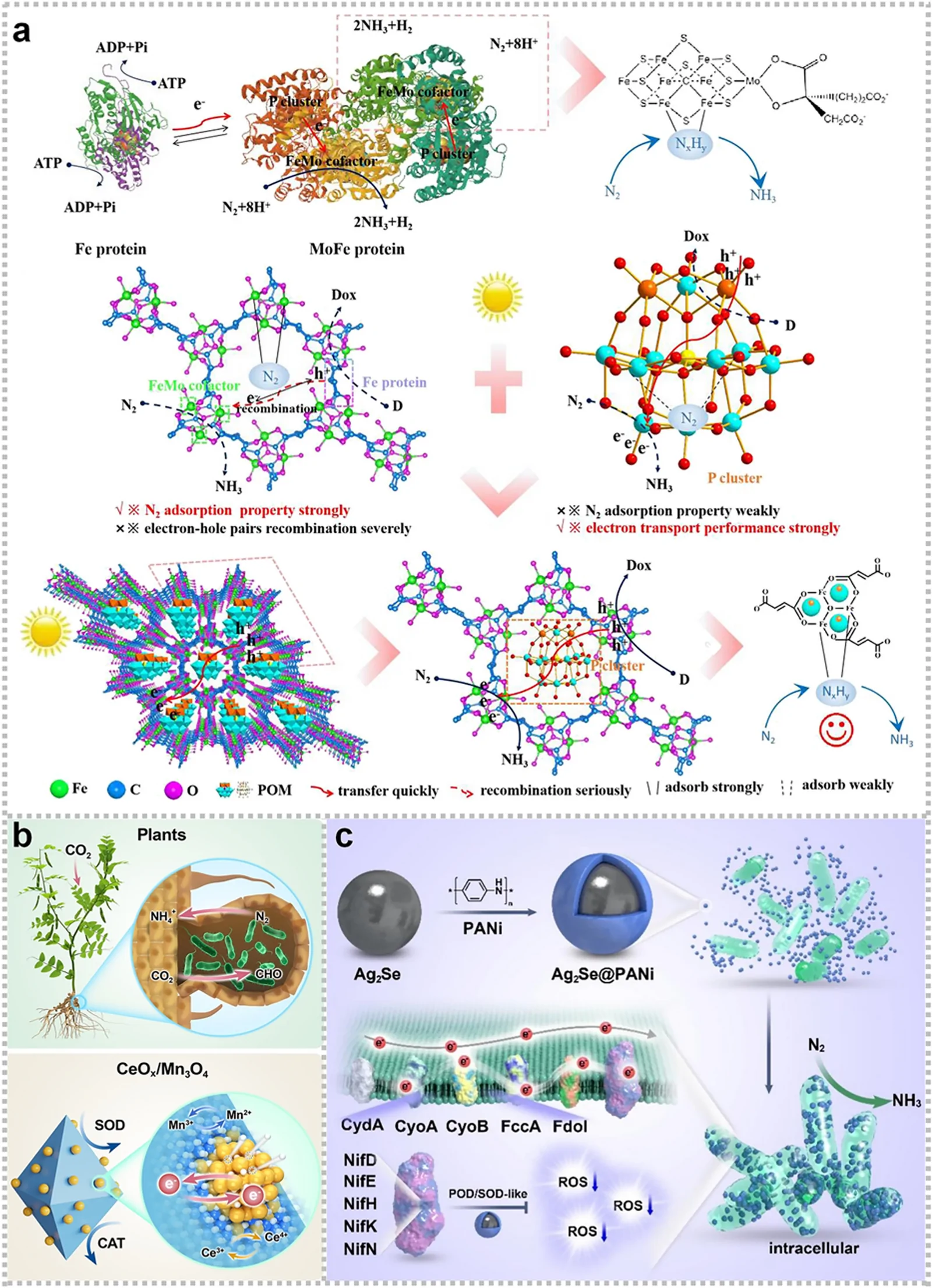
Nanozyme-mediated artificial nitrogen fixation and symbiotic nitrogen fixation. **a** Photocatalytic NH_3_ production over PMo_10_V_2_@MIL-88A via the π-back bonding mechanism inspired by nitrogenase. Reproduced with permission [52]. Copyright 2023, American Chemical Society. **b** Symbiotic nitrogen fixation between plants and microbes (top) and CeO_x_/Mn_3_O_4_ symbiotic nanozyme regulating Mn^3+^/Mn^2+^ and Ce^3+^/Ce^4+^ cycles via SOD- and CAT-like activities (bottom). Reproduced with permission [53]. Copyright 2025, Wiley–VCH. **c** Electron transfer mechanism and nitrogen fixation pathway of the Ag_2_Se@PANi core–shell nanozyme. Reproduced with permission [54]. Copyright 2025, Elsevier

*Nanozyme-mediated Artificial Nitrogen Fixation*: Nanozyme-mediated artificial nitrogen fixation has primarily focused on three strategies including material biomimicry, micro-nanostructure engineering, and photocatalysis to reconstruct electron transfer pathways and N≡N bond activation mechanisms. By leveraging multimetallic synergy, interfacial coupling, and photogenerated charge carrier modulation, these approaches have markedly enhanced NH_3_ production efficiency. In the realm of biomimetic materials, Ohki and colleagues constructed an Fe-Mo-S-C[Mo_3_S_4_Fe] cubane cluster [86], precisely mimicking the FeMoco active site. Spatially confined ligands expose the Fe sites and facilitate terminal N_2_ coordination, enabling efficient N_2_ reduction. By finely tuning the redox states of FeMoco, electron transfer capabilities are enhanced, thereby improving catalytic activity. In terms of nanostructure design, material engineering has been employed to achieve macroscopic electronic synergy and optimize interfacial active sites. Sun et al. modulated the electronic structure and active site distribution of MoSe_2_ via Fe doping, enhancing N_2_ adsorption activation while suppressing the hydrogen evolution reaction, achieving a production rate of 3.38 μg cm^−2^ h^−1^ with a faradaic efficiency of 30.8% [49]. Xu et al. constructed a MoO_2_/FeS_2_/graphene aerogel composite, where multiphase interfaces induce electronic synergy and optimize reaction kinetics, resulting in a nitrogen reduction reaction rate of 40.18 μg h^−1^ mg^−1^ and a faradaic efficiency of 37.44%. Wan et al. designed a nanozyme mimicking nitrate reductase and nitrite reductase structures (Cu NCs&SAs@CNFs)[51], where mesoporous carbon fibers create a confined microenvironment to enrich substrates and synergistically promote multi-electron/multi-proton transfer, achieving a production rate of 31.45 mg h^−1^ cm^−2^ and a Faradaic efficiency of 97.23%, while stably driving Zn-NO_3_^−^ batteries, providing a reference for microenvironmental regulation in biomimetic electrocatalytic systems. Additionally, photocatalytic strategies have been explored to enhance NH_3_ production by improving charge carrier separation and electron transfer efficiency. Xie et al. developed a metal–organic frameworks (MOFs)-polyoxometalates (POMs) composite photocatalytic platform (PMo_10_V_2_@MIL-88A) [52], where oxygen-rich POM units modulate the electronic states of MOF metals and mimic the π-feedback function of natural nitrogenases, significantly improving photogenerated carrier separation efficiency (Fig. 4a). This approach enabled the PMo_10_V_2_@MIL-88A system to reach an NH_3_ production rate of 50.82 μmol g^−1^ h^−1^, 6 and 14 times higher than single-component MIL-88A and PMo_10_V_2_, respectively.

Current nanozyme systems still have room for improvement in NH_3_ production rates and faradaic efficiencies, with limited structural stability and suboptimal photocatalytic activity. Most systems have been evaluated primarily under controlled laboratory conditions, and challenges such as catalytic stability, suppression of competitive hydrogen evolution, and scalability persist in practical applications. Moreover, the effects of environmental factors such as temperature, pH, and ionic strength on catalytic performance have not been systematically investigated. These limitations underscore the urgent need to develop systematic nanozyme platforms with high stability, environmental adaptability, and scalability to advance artificial nitrogen fixation toward efficient, sustainable, and practically deployable applications.

*Nanozyme-mediated Symbiotic Nitrogen Fixation*: Symbiotic nitrogen fixation represents a prototypical biologically coordinated and environmentally adaptive nitrogen cycling mode in nature. *Leguminous* plants and *Rhizobia* achieve efficient atmospheric nitrogen fixation through complex signaling recognition and regulatory networks, which are critical for soil nitrogen balance and sustainable agriculture. However, natural nitrogenase is highly oxygen-sensitive and prone to inactivation by ROS, limiting nitrogen fixation efficiency. Nanozymes, owing to their antioxidant capabilities, can efficiently scavenge ROS in the rhizosphere or within cells, protecting nitrogenase activity while simultaneously optimizing electron transfer and energy metabolism, thereby enabling multidimensional regulation of nitrogen fixation. Although research in this area remains exploratory, 11 relevant studies have established a framework centered on antioxidant protection, signal modulation, energy metabolism optimization, and host–symbiont interface engineering.

To address the challenge of irreversible nitrogenase inactivation caused by high ROS levels, Ma et al. developed an antioxidant cobalt ferrite (CoFe_2_O_4_) nanozyme [87], which protects nitrogenase by regulating ROS metabolism, resulting in a 260% increase in soybean nitrogen fixation efficiency. This nanozyme also reduces ROS levels in nodules, promotes rhizobial proliferation, and enhances hemoglobin accumulation, thereby increasing photosynthetic efficiency by 67.2%, demonstrating significant potential for enhancing symbiotic nitrogen fixation. Wu et al. further constructed a CeO_x_/Mn_3_O_4_ symbiotic nanozyme (Fig. 4b) [53], in which CeO_x_ is spatially anchored within the Mn_3_O_4_ lattice to form an interlaced lattice structure, enabling Mn-Ce electron transfer and precise valence state control, substantially improving electron transport efficiency and ROS scavenging capacity to protect nitrogenase activity. Zhao et al. developed an Ag_2_Se@PANi core–shell nanozyme, integrated into the *Paraburkholderia* Sb-24 system [54], which accelerates bacterial growth and lowers intracellular ROS levels to enhance nitrogenase activity (Fig. 4c). At 38 °C, the system achieved an NH_4_^+^ production of 0.48 ± 0.05 μg mL^−1^, representing an approximately 333% increase over the pure strain. Proteomic analysis revealed upregulation of Mo-Fe proteins and electron transfer-related proteins, confirming the critical role of interfacial electron transfer in improving nitrogen fixation efficiency.

Nanozyme-mediated symbiotic nitrogen fixation remains largely limited to antioxidant protection, with insufficient systematic development of multifunctional mechanisms such as reductive activity, multi-electron/proton coupling, and substrate selectivity, thereby constraining further improvements in overall nitrogen fixation efficiency. Moreover, challenges persist regarding the long-term performance, stability, environmental adaptability, and ecological safety of nanozymes. Future efforts should focus on developing nanozyme systems with multifunctional cascade activity to comprehensively enhance symbiotic nitrogen fixation and exploring low-cost production strategies to facilitate large-scale agricultural applications.

#### Nanozyme-Mediated Carbon Fixation

Carbon fixation is a key process in the global carbon cycle and ecosystem energy flow, involving enzymatic conversion of CO_2_ into organic carbon compounds. It is widely observed in plant photosynthesis, algal carbon-concentrating mechanisms, and microbial metabolism, providing essential carbon sources for biological systems. Recent studies have suggested that nanozyme-based strategies may regulate carbon fixation processes across different biological systems, with potential to enhance photosynthetic efficiency and microbial carbon assimilation, and to provide inspiration for applications in environmental remediation and agricultural production.

**Fig. 5:**
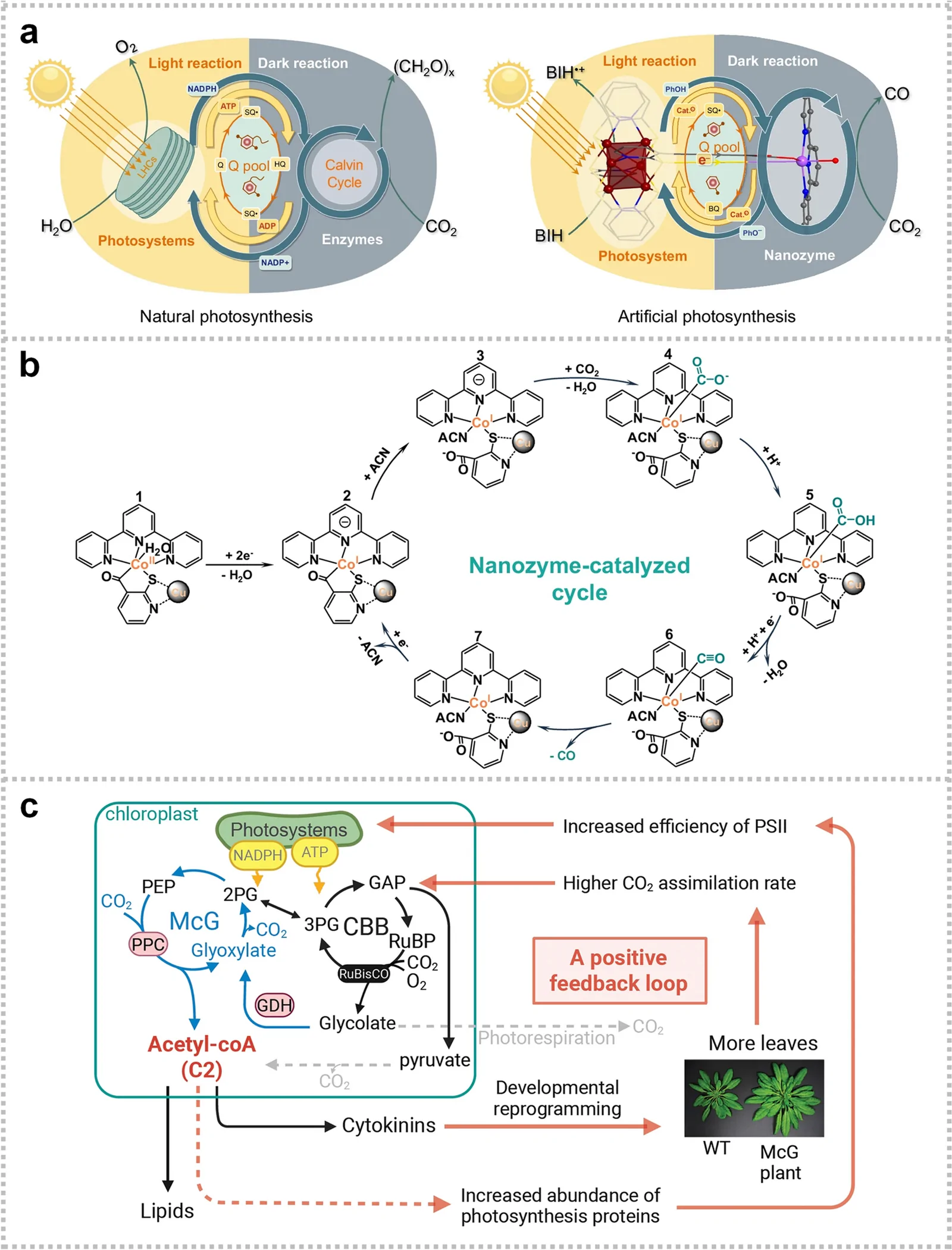
Nanozyme-mediated carbon fixation. **a** Schematic of natural photosynthesis (left) and artificial photosynthesis (right). **b** Mechanistic pathway of CO_2_ reduction catalyzed by Cu_6_-Cotpy. Reproduced with permission [55]. Copyright 2024, Springer Nature. **c** Dual-cycle, C_2_-centered carbon fixation system constructed in Arabidopsis. The McG cycle (blue) cooperates with the Calvin–Benson–Bassham (CBB) cycle (black) to enhance acetyl-CoA synthesis, bypass photorespiration, promote plant growth, and increase seed and lipid yields. Reproduced with permission [89]. Copyright 2025, American Association for the Advancement of Science

Natural photosynthesis relies on the precise coordination of light-harvesting complexes (LHCs), enzymes, and cofactors to efficiently convert water and CO_2_ into energy-rich carbohydrates. Inspired by this, Xiong et al. developed an artificial photosynthetic system composed of Cu_6_ nanoclusters and a cobalt-tris(2,2′-bipyridine) complex (Cu_6_-Cotpy, Fig. 5a) [55], in which the Cu nanoclusters serve as light-absorbing units and the molecular complex functions as a nanozyme catalytic center. Under the drive of photoexcited electrons, the Co sites sequentially mediate CO_2_ adsorption, *COOH formation, PCET dehydroxylation, and CO desorption, achieving efficient CO_2_ reduction to CO (Fig. 5b). This system exhibits a CO production rate of 740.7 μmol g^−1^ h^−1^, sustains catalysis for over 188 h, and decouples light and dark reactions to extend the dark reaction to 8.5 h, effectively mimicking diurnal cycles and enhancing the stability and energy efficiency of photocatalytic CO_2_ conversion. Building on efficient single-carbon product conversion, Meindl et al. developed a photocatalytic system centered on dPCN-224(H) MOF [88], employing photosensitive porphyrins as catalytic centers and leveraging ROS to achieve high-efficiency CO_2_ reduction to ethanol with a conversion efficiency of 92%. This system can be integrated with direct air capture (DAC) technology, enabling flexible utilization of gaseous or dissolved CO_2_ and establishing a versatile, sustainable solar-driven CO_2_ conversion strategy. To further enhance carbon fixation efficiency and optimize carbon flow in plants, researchers have explored introducing artificial carbon fixation cycles beyond native photosynthetic pathways, effectively coupling light absorption with carbon backbone synthesis. Following this approach, Lu et al. constructed the malonyl-CoA-glyoxylate (McG) cycle in *Arabidopsis* (Fig. 5c) [89], utilizing RuBisCO carboxylation and oxygenation products to generate acetyl-CoA, significantly boosting lipid synthesis and cytokinin levels. McG transgenic plants exhibited a twofold increase in CO_2_ assimilation under ambient CO_2_ conditions compared with wild-type, increased lipid accumulation in leaves and seeds, enhanced photosystem II quantum efficiency, 2–threefold increases in dry biomass, and a three-fold improvement in seed yield, collectively demonstrating substantial enhancement in overall photosynthetic performance and growth phenotypes.

By mimicking photosynthetic enzymes or introducing artificial carbon fixation cycles, CO_2_ conversion efficiency, product formation rates, and energy utilization can be significantly enhanced, thereby improving plant growth and metabolic flow. However, current carbon fixation strategies remain limited in precise control over product selectivity and CO_2_ utilization efficiency under complex environmental conditions. Future research should focus on constructing highly efficient, stable, and sustainable photocatalytic or in planta artificial carbon fixation systems, optimizing light absorption, electron transfer, and carbon metabolic pathways to enable multi-carbon product formation and realize industrial application potential.

#### Hydrogenase-Like Nanozymes

Hydrogen is the simplest and most abundant element in the universe and is widely regarded as an ideal energy carrier for future clean energy systems due to its high energy density and zero-carbon emission characteristics. Natural hydrogenases can efficiently catalyze H₂ production and activation under ambient conditions, and their precisely regulated proton–electron coupling transfer (PCET) mechanisms provide a biological paradigm for hydrogen energy conversion. Inspired by this, nanozymes have been developed to mimic hydrogenase-like catalytic activity, enabling efficient hydrogen transformation under mild conditions. In biomimetic design, Hu et al. inspired by the natural [Fe]-hydrogenase, constructed a Mn-substituted Fe active-site mimic complex to achieve H_2_ heterolysis and substrate hydrogenation reactions [90]. This Mn complex exhibited superior catalytic activity and stability compared to the natural enzyme, with a normalized activity enhancement of approximately 25%, demonstrating the high feasibility of Mn incorporation in biomimetic hydrogenases. Further, by tuning the ligand basicity and metal electronic configuration, the team designed octahedral Mn(I) complexes capable of mimicking the geometric and electronic features of natural enzyme active sites [91]. Leveraging the d^6^ electronic configuration of Mn(I) in concert with ligand cooperation, H_2_ activation performance was significantly optimized, with complexes containing 2-(*N*, *N*-dimethylamino) (2-NMe_2_) and carbene ligands showing an eightfold increase in catalytic activity compared to traditional 2-OH analogues, while achieving higher efficiency in natural substrate analog transformations. These results indicate that metal-center mimicking combined with ligand cooperativity can precisely replicate the PCET mechanism of natural hydrogenases, substantially enhancing hydrogenation activity. In multifunctional systems and external energy coupling design, Liang et al. developed NiCo_2_O_4_-based hydrogenase-like nanozymes, which were composited with Al-Sn alloys to form a NiCo_2_O_4_@Al-Sn system, enabling efficient H_2_ production in aqueous environments without external energy input [56]. This system constructs a tri-center Ni-OH-Co motif analogous to natural [NiFe]-hydrogenase to promote water splitting, achieving a hydrogen evolution rate of 915 L h^−1^ g^−1^, significantly surpassing the performance of the natural enzyme. He et al. synthesized OV-TiO_2_/Pd hybrid nanosheets (HNSs) via a one-step solid-state reduction, wherein the strong metal–support interaction (SMSI) effect between Pd and TiO_2_ and oxygen vacancies in TiO_2_ facilitate electron transfer from TiO_2_ to Pd NCs, reducing the Schottky barrier and forming a built-in electric field that enhances photogenerated charge separation and photocatalytic activity. Under illumination, this catalyst exhibits both hydrogenase- and OXD-like activities, pioneering a strategy for coupling photocatalysis with nanozyme catalysis [57].

Nanozymes can recapitulate the PCET mechanism of natural hydrogenases at the molecular level through metal-center mimicry and ligand synergistic design, thereby markedly enhancing H_2_ activation and substrate hydrogenation. The integration of external energy inputs (e.g., light, ultrasound, and magnetic fields) with multifunctional composite architectures can significantly enhance catalytic activity by promoting electron transfer, enhancing reactant mass transport, and regulating electron spin states. These strategies enable hydrogenase-oxidase functional coupling or facilitate efficient H_2_ generation under ambient temperature and pressure. Nevertheless, current systems still face limitations in mechanistic elucidation, multifunctional coupling efficiency, and long-term stability. Future efforts should focus on in situ characterization and interface engineering to optimize metal–ligand interactions, thereby advancing hydrogen energy conversion technologies toward high-efficiency and sustainable applications.

#### Methane Monooxygenase-Like Nanozymes

**Fig. 6:**
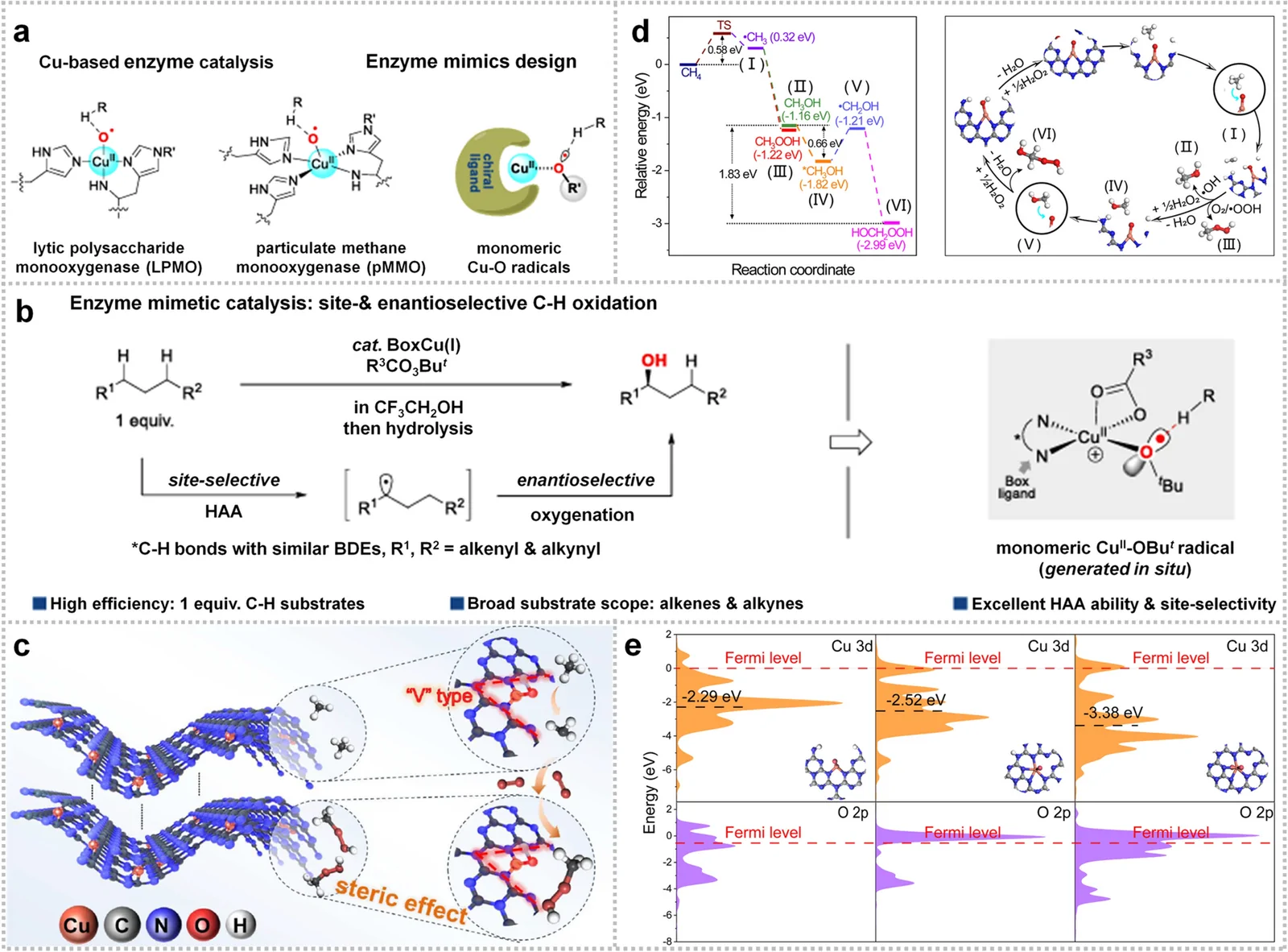
Methane monooxygenase-like nanozymes. **a** Copper-based MMO catalysis: structures of the active copper-oxygen intermediates in LPMO and pMMO, and the conceptual design of copper-based MMO mimics. **b** Cu(II)-tert-butylperoxide complex for asymmetric C(sp^3^)-H oxidation catalyzed by copper. Reproduced with permission [94]. Copyright 2025, Springer Nature. **c** Cu_1_/CN nanozyme catalyzing methane oxidation via H_2_O_2_ and O_2_ to generate highly reactive and selective oxygenated products. **d** Energy profile and reaction pathway diagram for methane conversion catalyzed by the Cu_1_/CN nanozyme. **e** Partial density of states (PDOS) analysis of CuN_2_O, CuN_4_O, and CuN_4_O_2_ sites. Reproduced with permission [58]. Copyright 2025, American Chemical Society

Efficient and sustainable selective oxidation of methane remains a major challenge in energy conversion. Methane monooxygenase (MMO), the only known biological catalyst capable of converting methane to methanol under ambient temperature and pressure, is widely regarded as an important biomimetic model for nanozyme design due to its copper-dependent regulation and highly efficient oxidative capability. Chan et al. noted that due to the inertness of the methane C-H bond [92], direct activation under mild conditions is challenging. The Cu^+^-Cu^+^-Cu^+^ tri-copper cluster at the D site of pMMO efficiently activates O_2_ to generate ROS and selectively converts methane to methanol via a direct insertion mechanism, providing a molecular basis for artificial system design. Building on this mechanism, researchers have developed controllable artificial systems to enhance enzyme stability and recyclability. Xin et al. generated Au nanoparticles in situ within pMMO-enriched membrane components and combined them with MMO-Cu (Mb-Cu) to form a nano-biohybrid system [93], significantly enhancing pMMO activity and stability. In this system, Mb-Cu binds to AuNPs via Au–S bonds and interfaces with pMMO, restoring electron transfer and protecting the enzyme from ROS, enabling hydroxyl quinone-driven quantitative methane conversion and providing a molecular foundation for nanozyme design. Copper-based MMOs, such as lvtic polysaccharide monooxygenase (LPMO) and pMMO, utilize Cu–O radical intermediates to efficiently abstract hydrogen atoms from strong C-H bonds, driving highly selective C(sp^3^)-H oxidation (Fig. 6a). Inspired by this mechanism, Zhang et al. developed a biomimetic Cu(II)-tert-butylperoxide catalytic system that achieves efficient asymmetric C(sp^3^)-H oxidation even under substrate-limited conditions (Fig. 6b) [94], demonstrating remarkable site selectivity and stereocontrol. This strategy provides a biomimetic catalytic pathway for late-stage C-H functionalization of complex bioactive molecules. Despite the high selectivity of copper-based biomimetic systems, the high bond dissociation energy of methane C-H bonds still limits activity. To overcome this bottleneck, Kojima et al. designed an Fe^2+^-*N*-heterocyclic carbene (NHC) nanozyme, introducing a hydrophobic secondary coordination sphere on the pyridine ring to mimic the hydrophobic pocket of natural enzymes [95], thereby enriching and activating methane. This system generates Fe^4+^ = O active species via PCET, and C-H cleavage followed by oxygen rebound forms methanol through a hydrogen tunneling mechanism, substantially lowering the reaction barrier and achieving an 83% conversion rate. Zhang et al. developed a biomimetic Cu SAzymes (Cu_1_/CN, Fig. 6c) [58], where the activation energy barrier for methane C-H bond cleavage is only 0.58 eV. The resulting methyl radicals couple with O_2_ to form oxygenated products such as methyl hydroperoxide, achieving a turnover frequency (TOF) of 405.3 ± 8.2 h^−1^. The “V”-shaped coordination structure around the Cu_1_ active site sterically suppresses the back diffusion of CH_3_OOH to the Cu–O center, achieving near 100% selectivity. Furthermore, N_2_-Cu_1_-OH can be oxidized by •OH to form N_2_-Cu_1_-O. The covalent character of the Cu–O bond (Fig. 6d) and the low d-band center (− 2.29 eV, Fig. 6e) facilitate charge transfer with methane molecules, enhancing reactivity in direct methane conversion (DMC) reactions. While the preceding studies focused on thermochemical biomimetic catalysts for selective methane activation, Li et al. developed a biomimetic photocatalytic system by regulating the spatiotemporal migration of photogenerated charge carriers in platinum-loaded cadmium sulfide (Pt/CdS) [96], thereby mimicking the ordered two-step catalytic mechanism of MMO. This strategy enables the spatial decoupling of hydroxyl radical generation from methane dehydrogenation, effectively suppressing methanol overoxidation and ultimately achieving a methane-to-methanol selectivity of 83.5%.

By mimicking the active sites of pMMO and its electronic and spatial microenvironment, nanozymes can achieve highly selective oxidation of methane C-H bonds under mild conditions. Through combined strategies of electronic transfer restoration, hydrophobic microenvironment construction, and generation of highly reactive intermediates, controlled conversion of methane to methanol or asymmetric C-H oxidation products can be realized. Concurrently, the design of nanozyme photocatalytic systems with precise spatiotemporal control of photogenerated charge carriers enables significant enhancement of methane oxidation selectivity and catalytic efficiency. Nevertheless, current systems remain limited by catalytic activity, substrate dependence, and stability. Future efforts should focus on precise active site engineering, enhanced substrate enrichment, and synergistic driving strategies to achieve efficient and sustainable methane oxidation.

### Nanozyme-mediated Energy Conversion Devices

Nanozymes precisely regulate catalytic active sites through biomimetic strategies, enabling highly efficient coupling and transport of electrons, protons, and reactants in energy conversion processes, thereby optimizing reaction kinetics and enhancing system stability. Compared with natural enzymes, nanozymes exhibit superior structural stability and environmental adaptability, maintaining stable catalytic activity across wide ranges of temperature, pH, and electrolyte conditions. Through the multiscale construction of single-atom, metal-cluster, and composite nanostructures, nanozymes can establish enzyme-like interfacial architectures and synergistic transport networks, enabling cross-scale regulation of energy and mass transfer from the molecular level to device systems. In energy storage and conversion systems, this strategy simultaneously enhances reaction kinetics, interfacial stability, and cycling reversibility, while also expanding functional versatility. Within the eco-nanozymology framework, fuel cells and meta–air batteries can be further regarded as biomimetic energy transduction nodes, thereby providing a unified theoretical basis and design paradigm for Li–O_2_ batteries, Li–S batteries, Zn–air batteries, and BFCs.

#### ***Li–*** O_***2***_*** Batteries***

LiO_2_ is a key intermediate in the ORR of Li–O_2_ and Li–air batteries, and its dissolution can trigger side reactions that degrade battery performance. Therefore, controlling the formation and reaction pathways of LiO_2_ is critical for enhancing battery efficiency. Inspired by SOD, Ren et al. incorporated oxidatively active carbon (OAC) as an SOD mimic into a dual-polymer gel electrolyte [97], improving battery stability. The OAC-enhanced Li–O_2_ battery retained a discharge capacity of ∼ 37.0 mAh cm^−2^ and a cutoff capacity of 0.4 mAh cm^−2^ even after 300 charge–discharge cycles, significantly extending cycle life. Wang et al. introduced the copper(I) complex 3 N-Cu^I^ into Li-O_2_ batteries to modulate the reaction pathway [98], enabling the reversible cleavage and formation of O–O bonds at room temperature. The 3 N-Cu^I^ catalyzed a four-electron O_2_ reduction to LiOH via a dicopper-dioxygen intermediate, suppressing superoxide side reactions, stabilizing the carbon cathode, and markedly improving specific capacity, lowering overpotentials, and extending cycle life. To further enhance ORR/OER kinetics and stability, bioinspired designs have emerged as a key strategy. Mu et al. incorporated a MOF nanozyme (Zn-TCPP(Fe)) [59], constructed from tetra(para-carboxyphenyl)porphyrin iron ligands and zinc nodes, into the Li-O_2_ electrolyte as a bifunctional catalyst. During both discharge and charge, this system coordinates with O_2_^•−^ intermediates to facilitate efficient electron and O_2_^•−^ transport between the cathode and Li_2_O_2_, significantly enhancing discharge capacity, energy efficiency, and cycle stability. On this basis, Li et al. further optimized a heme-like system by constructing a MOF nanozyme (Co-TCPP(Fe)), composed of TCPP(Fe) ligands and cobalt nodes [99]. Leveraging the highly ordered channels and robust adsorption–activation properties of the MOF structure, Co-TCPP(Fe) enables synergistic radical scavenging and superoxide shuttle catalysis, thereby accelerating Li_2_O_2_ formation/decomposition kinetics, increasing discharge capacity by ∼67%, and nearly doubling cycle life.

Nanozymes can significantly enhance the discharge capacity and cycling stability of Li–O_2_ batteries by optimizing the kinetics of the ORR and the Li_2_O_2_ formation/decomposition processes. However, challenges remain regarding their long-term stability, electrolyte compatibility, and further efficiency improvements. Future efforts should focus on optimizing nanozyme structures and electrolyte systems to further boost battery performance and extend operational lifespan.

#### Li–S Batteries

**Fig. 7:**
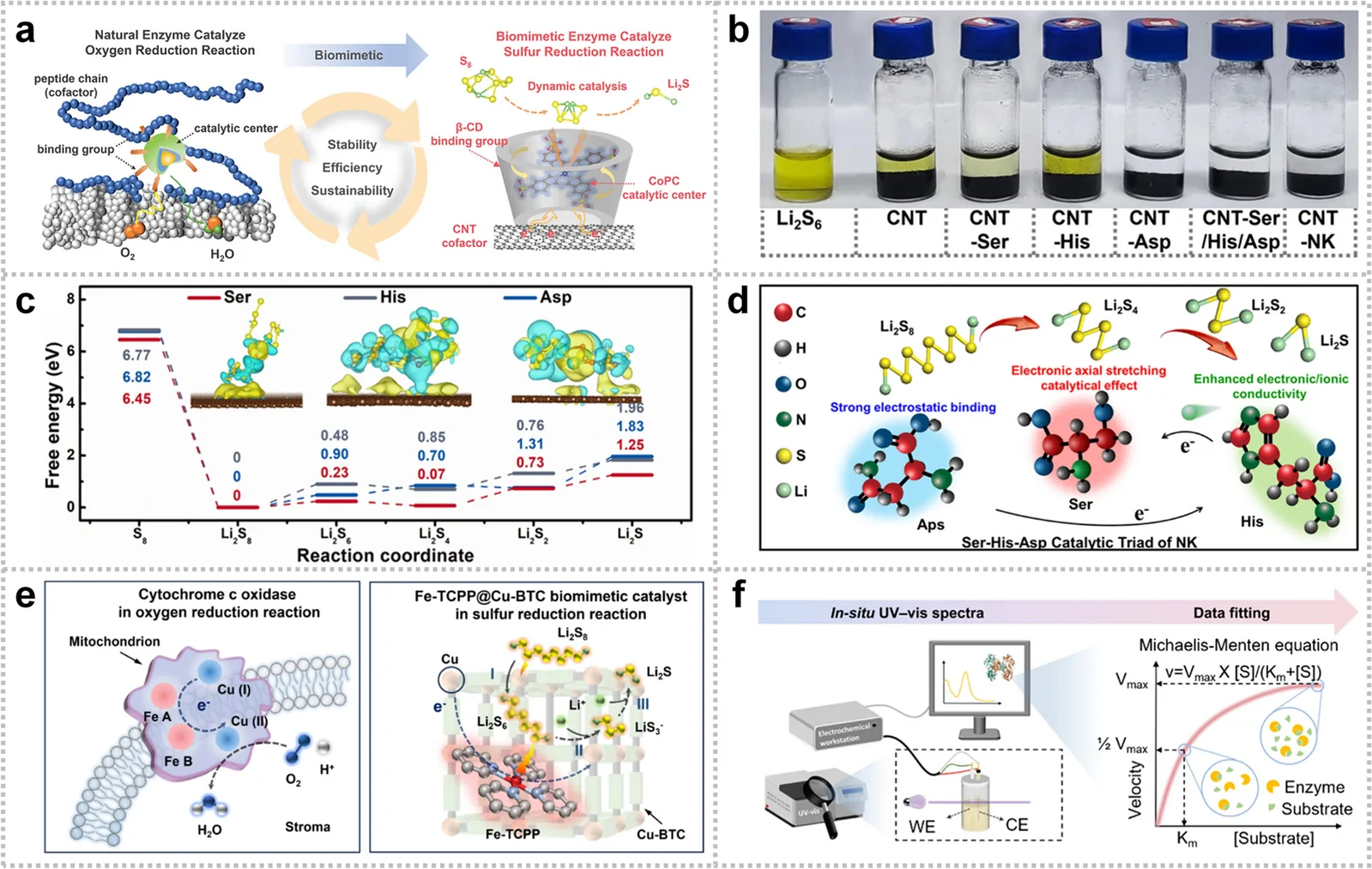
Nanozyme-mediated Li–S batteries. **a** Comparison of the catalytic centers in natural enzymes and the β-CD@CoPC nanozyme. Reproduced with permission [60]. Copyright 2024, Wiley–VCH. **b** Photographs showing the color changes of different CNT-based materials in Li_2_S_6_ solution after 2 h. **c** Gibbs free energy profiles for the S_8_-to-Li_2_S reduction on Asp, Ser, and His substrates, along with the charge density differences of the three surfaces interacting with Li_2_S_8_. Yellow (blue) regions indicate charge accumulation (depletion). **d** Schematic illustration of the catalytic chemical reactions in Li–S batteries, regulated by the Ser-His-Asp triad. Reproduced with permission [61]. Copyright 2025, American Chemical Society. **e** Fe-TCPP@Cu-BTC nanozyme designed for Li–S batteries, inspired by the catalytic structure of CcO. **f** Schematic of in situ UV–vis spectroscopic identification of different LiPS species during Fe-TCPP@Cu-BTC catalysis. The working electrode and reference electrode are abbreviated as working electrode (WE) and reference electrode (RE), respectively. Reproduced with permission [62]. Copyright 2025, American Chemical Society

Despite the high theoretical energy density of Li–S batteries, their practical performance is largely limited by sluggish sulfur redox reaction (SRR) kinetics, especially under high sulfur loading and lean electrolyte conditions. To address the sluggish SRR kinetics under high sulfur loading and low E/S ratios, Dong et al. embedded sulfonated cobalt phthalocyanine (CoPC) into the cavity of β-cyclodextrin (β-CD) to construct a β-CD@CoPC nanozyme [60], mimicking the active centers of natural sulfur oxidases or peroxidases. The dynamic stereo conformation of the hydrophobic β-CD cavity enables flexible encapsulation and release of CoPC active sites and Li_2_S intermediates, facilitating sulfur redox reactions and Li_2_S separation (Fig. 7a). Michaelis–Menten analysis showed that its catalytic activity is approximately three- and 30-fold higher than that of β-CD and CoPC, respectively. Under high-sulfur loading (12.8 mg cm^−2^) and low E/S ratio (3.5 µL mg^−1^), Li–S batteries achieved an initial areal capacity of 11.6 mAh cm^−2^, providing an effective strategy for sulfur conversion under lean electrolyte conditions. To further enhance sulfur cascade conversion efficiency and high-rate performance, Wang et al. drew inspiration from the thrombolytic and microcirculation functions of the peptide-based nattokinase (NK) [61], developing NK and peptide-like enzymes (serine-histidine-aspartic acid (Ser-His-Asp) triad active center) as catalysts. Carbon nanotube (CNT)-Asp and CNT-Ser-His-Asp rapidly rendered Li_2_S_6_ solutions transparent, indicating strong adsorption capability (Fig. 7b). Among these, Ser exhibited the lowest energy barrier during Li_2_S_8_ reduction to Li_2_S, demonstrating its thermodynamic advantage in lithium polysulfides (LiPS) catalytic conversion (Fig. 7c). Additionally, Ser facilitates sulfur cascade conversion via electron axial stretching catalysis, Asp through strong anionic adsorption, and nitrogen-rich His via Li-affinity, synergistically promoting sulfur redox (Fig. 7d). After machine learning (ML)-guided formulation optimization, Li–S cells achieved a high-rate capacity of 744 mAh g^−1^ at 5 C and excellent cycling stability at 0.5 C, with an aerial capacity of 8.87 mAh cm^−2^ under E/S = 4 µL mg^−1^. To tackle slow sulfur conversion and severe shuttle effects, Li et al. were inspired by cytochrome c oxidase (CcO) to construct a spatially confined nanozyme (Fe-TCPP@Cu-BTC) [62], mimicking Cu/Fe active sites to promote Li_2_S_6_ decomposition and Li_2_S formation (Fig. 7e). Steady-state kinetics analyzed via in situ UV–vis spectroscopy (Fig. 7f) showed that Fe-TCPP@Cu-BTC exhibits *K*_*m*_ values of 5.92 × 10^–3^ and 7.79 × 10^–4^ mM and *V*_max_ values of 5.87 × 10^–4^ and 1.87 × 10^–4^ mM min^−1^ in the 2.8–2.2 and 2.1–1.6 V ranges, respectively, all significantly higher than those of Fe-TCPP or Cu-BTC. By using oxidoreductase-like catalysis, Fe-TCPP@Cu-BTC substantially accelerates sulfur conversion, enabling high-performance Li–S batteries with excellent cycling stability and energy density exceeding 380 Wh kg^−1^. Building on this, Guo et al. proposed embedding the nanozyme Co-B_1_N_3_ into microreactors (CoB_1_N_3_-MR) and constructing modular electrodes (CoB_1_N_3_-MR/FNN) [100]. The nanozyme mimics intracellular enzyme-like processes of capture, localization, binding, and conversion to accelerate sulfur redox reactions. Coupled with a neural network-inspired modular architecture, this design achieves multi-pathway charge transport and system-level fault tolerance, thereby markedly improving reaction efficiency, specific capacity, and rate performance.

Nanozymes enhance the kinetics of ORR and Li_2_O_2_ formation/decomposition in Li–O_2_ batteries through multiple mechanisms, including stabilization of reaction intermediates, provision of high-density, multi-electron active sites, radical scavenging, and optimization of electron and ion transport pathways. Collectively, these effects suppress side reactions, thereby improving the battery’s specific capacity, energy efficiency, and cycling stability. Nevertheless, the limited durability, selectivity, and incomplete mechanistic understanding of nanozymes remain critical challenges. Future efforts should focus on multiscale synergistic design and intelligent regulation to further optimize performance, enabling highly efficient and controllable Li–S battery systems.

#### Zn–Air Batteries

Zn–air batteries, regarded as next-generation energy storage systems due to their high energy density, low cost, and inherent safety, suffer from sluggish ORR/OER kinetics at the air cathode and side reactions caused by intermediates such as H_2_O_2_. Studies have shown that single-atom and carbon-based nanozymes, by mimicking natural enzyme active sites, can enhance catalytic activity, reduce overpotentials, and improve cycling stability. Xie et al. designed a biomimetic covalent organic framework (COF) based on CcO [63], using iron phthalocyanine (FePc) as the building block and incorporating quaternary ammonium ions to create a bio-inspired channel environment. The quaternized COF possesses a high density of Fe active sites, excellent conductivity, and structural stability, exhibiting a half-wave potential of 0.94 V (vs RHE) in ORR and achieving a power density of 211.5 mW cm^−2^ in Zn–air batteries. To further optimize catalytic activity, the researchers introduced the electronic structure of natural enzyme metal active sites to improve reactant adsorption and activation. Jiang et al. proposed the incorporation of single-atom copper into Fe-N-C biomimetic catalysts [64], developing CuN_5_-assisted Fe-N_5_ SAzymes (SA-Fe-N_5_). Electrochemical tests show that SA-Fe-N_5_ achieves a half-wave potential of 0.88 V (vs RHE) in alkaline solution, with a diffusion-limited current density of 5.9 mA cm^−2^, comparable to that of commercial Pt/C. Theoretical calculations indicate that the copper atom enhances the electron density of the iron sites through electron donation, optimizing the adsorption and desorption energies of ORR intermediates. In Zn–air batteries, SA-Fe-N_5_ demonstrates a power density of 217.8 mW cm^−2^ and a current density of 257.3 mA cm^−2^.

Overall, by biomimicking the natural enzyme structure and bio-inspired channels to precisely regulate active sites and electron/proton transport, nanozymes can significantly enhance the electrocatalytic performance of Zn–air batteries. However, challenges remain, including difficulty in precisely controlling the active center, limitations in optimizing the electronic structure, and insufficient regulation of intermediate products. Future research could focus on multi-metallic synergy or heterogeneous structure design, optimization of electron/proton transport channels, and utilizing ML for intelligent interface microenvironment regulation, thereby constructing efficient, stable, and scalable Zn–air batteries catalytic systems with great application potential.

#### Biofuel Cells

**Fig. 8:**
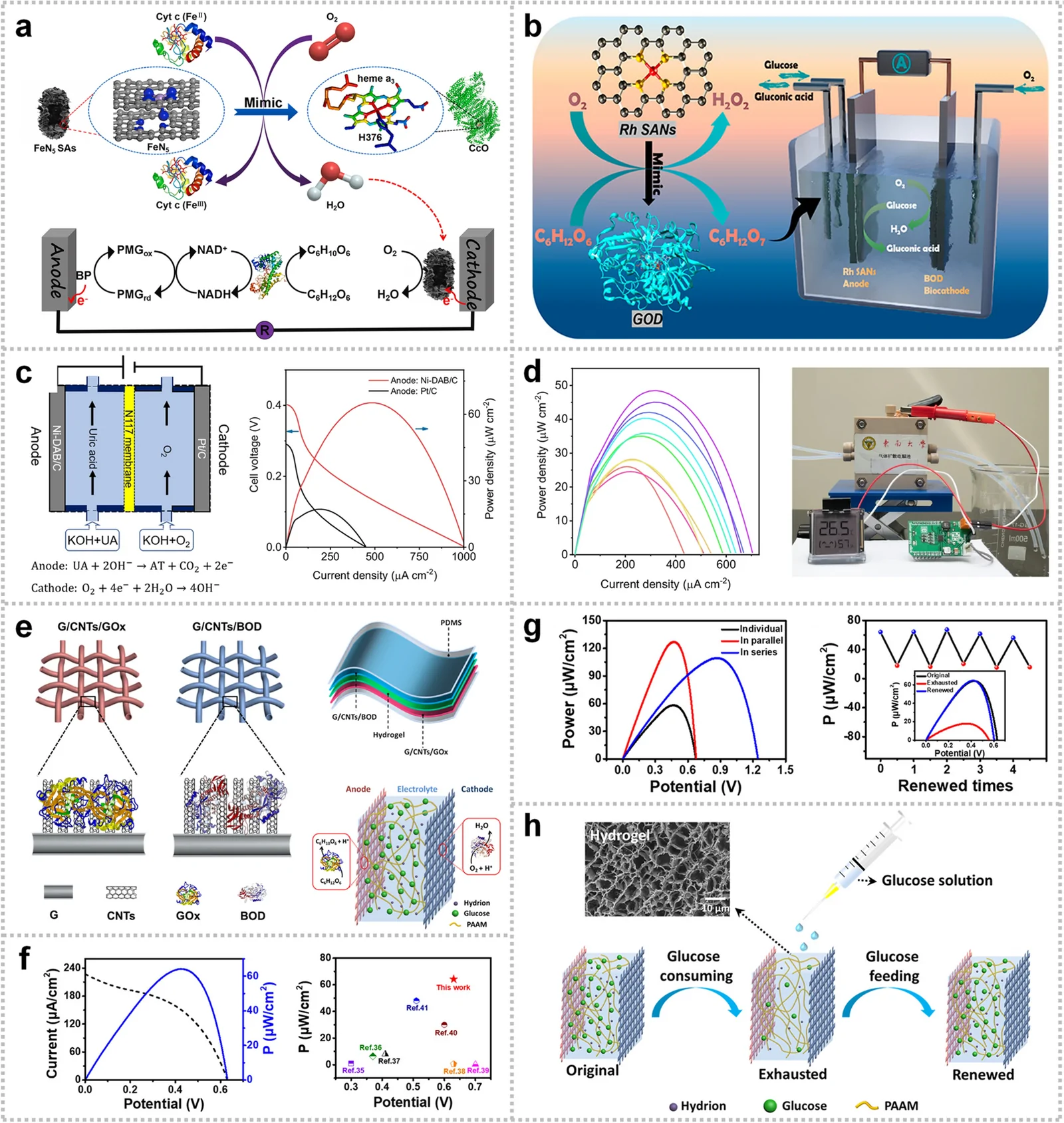
Nanozyme-mediated biofuel cells. **a** Structural and catalytic mechanism schematic of the FeN_5_ SAzyme and natural CcO, together with a schematic illustration of a glucose/O_2_ EBFC. Reproduced with permission [65]. Copyright 2021, Elsevier. **b** Rh SANs rationally designed based on the catalytic function of natural GOx, and their application in glucose/O_2_ EBFCs. Reproduced with permission [66]. Copyright 2023, American Chemical Society. **c** Schematic illustration of the BFC@Ni-DAB/C device and a comparison of the polarization curves and power density curves between BFC@Ni-DAB/C and BFC@Pt/C. **d** Power density curves of BFC@Ni-DAB/C using urine as the source of UA, and its application in driving a temperature-humidity sensor. Reproduced with permission [101]. Copyright 2024, Springer Nature. **e** Schematic illustration of G/CNTs/enzyme composite textile bioelectrodes and flexible, stretchable biofuel cells. **f** Power output curve (blue) and polarization curve (black) of the biofuel cell, together with a comparison of power output density and open-circuit voltage between the enzymatic biofuel cell and other previously reported systems. **g** Power curves of two biofuel cells connected in parallel (red curve) and in series (blue curve), along with regenerative performance tests over four cycles (inset: power curve of a single cycle). **h** Schematic illustration of the regenerative process of the biofuel cell. Reproduced with permission [67]. Copyright 2022, American Chemical Society

BFCs use natural organic compounds as fuel and are characterized by their renewability, mild reaction conditions, environmental friendliness, and good biocompatibility, making them suitable for wearable and implantable devices. Inspired by natural CcO, Zhang et al. developed an axial nitrogen-coordinated FeN_5_ SAzymes (FeN_5_ SAs) to mimic the heme a_3_ oxygen-binding site [65], enabling cytochrome c oxidation and ORR (Fig. 8a). This system demonstrated a half-wave potential of 0.67 V (vs. RHE) under neutral conditions and, when coupled with glucose dehydrogenase (GDH), constructed an EBFC, achieving a maximum power density of 149.2 ± 4.0 μW cm^−2^ and an open-circuit potential of 0.40 ± 0.01 V. This provides a strategy for SAzymes design inspired by natural enzymes and energy conversion. To improve catalytic efficiency and substrate selectivity, Zhao et al. developed a Rh SAzymes (Rh SANs) mimicking the catalytic activity of glucose oxidase (GOx) [66], achieving high alignment between glucose biocatalysis and two-electron electrochemical oxidation pathways. They subsequently constructed a Rh SANs/bilirubin oxidase (BOD) coupled EBFC (Fig. 8b), achieving a maximum power density of 135.0 ± 3.0 μW cm^−2^, significantly outperforming natural GOx systems, showing the potential of nanozymes in portable bioenergy devices. Considering the challenge of insufficient substrate selectivity in complex bodily fluids, Zhang et al. designed a SAzymes that mimics uric acid (UA) oxidation [101], incorporating metal–ligand dual active sites into the catalytic center, enabling high specificity recognition and electron transfer for UA. Based on this nanozyme, a BFC was developed (Fig. 8c) capable of generating electricity from human urine (Fig. 8d), expanding the application of BFCs in implantable medical devices and self-powered systems for extreme environments. Despite significant advancements in catalytic efficiency and substrate selectivity, challenges remain for the application of SAzymes in flexible, wearable, and stretchable bioenergy devices. Chen et al. proposed a flexible, stretchable BFC based on polymer hydrogel electrolytes (Fig. 8e) [67], utilizing textile-like covalently connected graphene/carbon nanotube (G/CNTs) composite electrodes to immobilize GOx and BOD, enabling efficient electron transfer and glucose oxidation–reduction energy conversion. This design maintained stable performance under 60% stretching and arbitrary bending, with an open-circuit voltage of 0.65 V and a maximum power density of 64.2 μW cm^−2^, outperforming most reported enzyme-based BFCs (Fig. 8f). Moreover, two BFCs connected in series could achieve a 1.25 V output voltage, effectively overcoming the voltage limitation of a single BFC. Connecting in parallel increased the maximum power density to 127.3 μW cm^−2^, twice that of a single BFC (Fig. 8g). Additionally, the porous structure of the hydrogel allows glucose solution to be efficiently injected via syringe, with the output power density recovering to 87.7% of its initial value (Fig. 8h), demonstrating its potential for flexible wearable electronic devices.

Through the biomimetic strategy of nanozymes, the energy density, catalytic efficiency, and multi-signal output capabilities of BFCs have been significantly enhanced, providing a new pathway for powering flexible, self-powered biosensors and wearable devices. However, challenges remain in terms of stability, substrate selectivity, system integration, and commercialization. Future research should focus on improving the stability of nanozymes, optimizing substrate selectivity and reaction efficiency, enhancing system functionality integration, and advancing large-scale applications to achieve widespread commercialization.

### Nanozyme-Mediated Biomass Valorization

Within the framework of “dual carbon” targets and a circular bioeconomy, nanozymes have emerged as biomimetic catalysts for the selective transformation of complex organic wastes under mild conditions [102]. Lignin, the most abundant aromatic biopolymer in biomass, possesses a highly cross-linked structure that limits conventional depolymerization. In addition, nanozyme-mediated conversion of recalcitrant biomass in composting systems can promote humic substance formation and enhance soil carbon sequestration, linking biomass utilization with ecological sustainability.

**Fig. 9:**
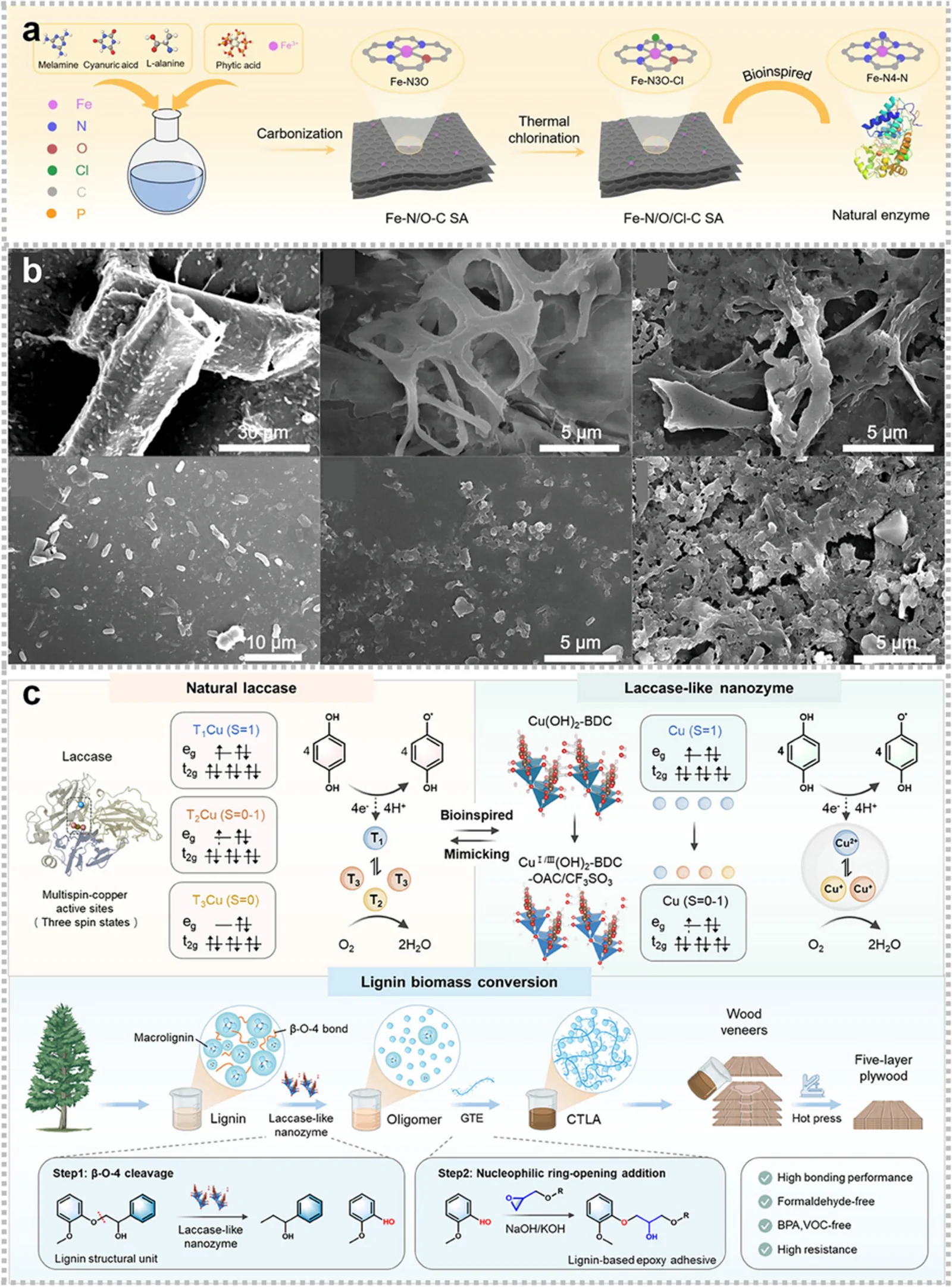
Nanozyme-mediated lignin degradation and valorization. **a** Schematic illustration of the synthesis of the Fe–N/O/Cl–C SAzyme. Reproduced with permission [69]. Copyright 2025, American Chemical Society. **b** SEM images and corresponding high-resolution images of corn stover treated with ε-MnO_2_ at 0 °C for different durations (0, 7, and 14 days). Reproduced with permission [104]. Copyright 2025, Wiley–VCH. **c** Inspired by the multispin-state characteristics of natural laccase, a two-dimensional copper-based MOF, denoted as COHBLO, was developed [71]. Copyright 2026, Wiley–VCH

To overcome the intrinsic limitations of natural enzymes in terms of stability and multivalent oxidative capability, a variety of nanozyme design strategies have been proposed. In the context of MOF nanozymes (MOFzymes), Zhou et al. designed a Cu/GMP-MOFzyme in which Cu^+^/Cu^2+^ multicopper clusters were constructed in situ within the framework and further modified with L-cysteine to form biomimetic catalytic pockets, thereby mimicking the copper-centered active environment of natural laccases [68]. This system enabled efficient cleavage of β-O-4 linkages and aromatic lactone bonds at room temperature, affording lignin oligomers with a high yield of 81.7 wt%, while exhibiting catalytic rates and stability superior to those of native enzymes. For SAzymes, Li et al. introduced axial chlorine coordination into Fe-N_3_O single-atom sites to construct an Fe-N/O/Cl-C SAzyme (Fig. 9a) [69]. The resulting five-coordinated configuration provided multiple exposed active sites, enabling parallel multivalent oxidation of lignin and delivering a catalytic efficiency (*K*_cat_*/K*_*m*_) of 1.75 × 10^7^ M^−1^ min^−1^, which is 1.96-fold higher than that of natural HRP. Dual-site biomimetic designs have further enhanced catalytic efficiency and multivalent reactivity. Qiu et al. developed Pd@Ce-PZDC, in which activated Ce(IV) sites simultaneously mimic LAC- and POD-like functions [103], thereby effectively promoting birch lignin depolymerization with catalytic activity surpassing that of native laccase. Multienzyme cascade strategies have also proven highly effective in lignin degradation. Fei et al. constructed a hybrid system comprising CotA laccase and λ-MnO_2_ nanozyme (CotA + λ-MnO_2_) [70], achieving a degradation efficiency of 25.15% and an aromatic product fraction of 48%. These results highlight the feasibility of cascade catalysis involving LAC-mediated demethoxylation and •OH/O_2_^•−^ radical-driven oxidation for efficient lignin depolymerization. Moreover, to address kinetically limited catalysis under low-temperature conditions, Tian et al. proposed a cold-adaptive MnO_x_-based nanozyme strategy. By systematically comparing different oxidation states and crystal phases, ε-MnO_2_ was identified as exhibiting optimal low-temperature activity over a wide range from -20 to 45 °C (Fig. 9b) [104]. Mechanistic investigations revealed that high-spin Mn(III) facilitates O–O bond dissociation through Jahn–Teller distortion, thereby lowering the reaction energy barrier and providing a new paradigm for the mild, low-temperature oxidation of straw biomass. More recently, Yu et al. reported a two-dimensional copper-based MOF nanozyme (COHBLO) design strategy based on copper spin-state modulation (Fig. 9c) [71]. Through precise control of redox processes and ligand exchange, copper sites with distinct spin states were engineered, revealing a volcano-type relationship between spin magnetic moment and LAC-like activity. This approach yielded a highly efficient COHBLO nanozyme with an activity 5.14 times higher than that of natural laccase. Notably, this work established a complete technological route from controllable lignin depolymerization to the fabrication of environmentally friendly COHBLO-treated lignin adhesive (CTLA) adhesives, thereby demonstrating a viable pathway for the high-value valorization of lignin.

Humic substances are stable carbon-rich components of soil that contribute to soil organic matter, structure improvement, and carbon sequestration. Enhancing their formation during composting is a key challenge for agricultural waste valorization. To address the recalcitrance of lignocellulose and low humification efficiency, nanozymes mimicking natural oxidases have been explored to improve redox activity and catalytic performance in composting systems. Liu et al. combined ball-milled manganese dioxide nanozymes (MDMP) with *Rhodococcus* sp. ZY-2 in aerobic rice straw composting [105]. ZY-2 decomposes organic matter to generate humic substance precursors, including asphaltol and quinones, while MDMP catalyzes their polymerization into stable humic acids via oxygen vacancies and modulates the microbial community to reduce greenhouse gas emissions. This approach leverages synergistic interactions between microbes and nanozymes to improve lignocellulose degradation and promote the polymerization of humic small molecules. Likewise, Lu et al. developed FeS_2_/Pal composites by modifying palygorskite with FeS_2_ [72], which exhibit glutathione OXD- and POD-like activities, thereby enhancing lignocellulose depolymerization and the polymerization rate of small humic molecules, ultimately improving compost maturity and humic substance polymerization. On the other hand, to maintain the long-term activity of natural enzymes under composting conditions, Koyani et al. encapsulated laccase in chitosan-tripolyphosphate nanoparticles [73], achieving high encapsulation efficiency and significantly improving enzyme stability and sustained catalytic activity, which in turn enhances humic substance formation efficiency.

Through precise structural modulation, rational engineering of metal centers, and synergistic integration of multienzymatic functionalities, nanozymes effectively address the inherent limitations of natural enzymes in lignin and lignocellulose degradation, including insufficient catalytic stability, restricted active-site selectivity, and limited multivalent oxidative capability, thereby markedly enhancing depolymerization efficiency, substrate conversion, and humic substance formation. Advances in cold-adaptive nanozymes, together with encapsulation and surface-functionalization strategies, further demonstrate that electronic structure and crystal-phase engineering can sustain high catalytic performance and prolonged activity under complex environmental conditions, including composting. Nonetheless, challenges remain regarding limited active site density, suboptimal substrate specificity, long-term operational stability, synergistic mechanism optimization, and industrial scalability. In particular, structural robustness under complex conditions, high production costs, and inefficient recovery and recycling continue to constrain economic and practical feasibility. Future efforts should prioritize the integration of multifunctional active sites, optimization of electron-transfer pathways, intelligent surface functionalization, rational multienzyme cascade architectures, and the development of cost-effective, recyclable nanozyme systems to realize high-performance, durable, and engineerable platforms for lignin and lignocellulose valorization, humic substance formation, and greenhouse gas mitigation, while enabling scalable and sustainable utilization of agricultural wastes.

### Nanozyme-Mediated Environmental Pollutant Remediation

Organic pollutants, pesticide residues, and microplastics are persistent contaminants in environmental systems, characterized by stability, recalcitrance, and bioaccumulation, leading to long-term risks to ecosystems and human health [106, 107]. Conventional physicochemical treatment methods suffer from low selectivity and limited efficiency, while natural enzymes are constrained by poor stability and high cost. Nanozymes, as biomimetic alternatives, offer tunable active sites and improved stability, enabling efficient catalytic degradation of persistent pollutants by mimicking oxidase or hydrolase functions and regulating electron-transfer pathways, providing promising strategies for environmental remediation.

#### Organic Pollutants

**Fig. 10:**
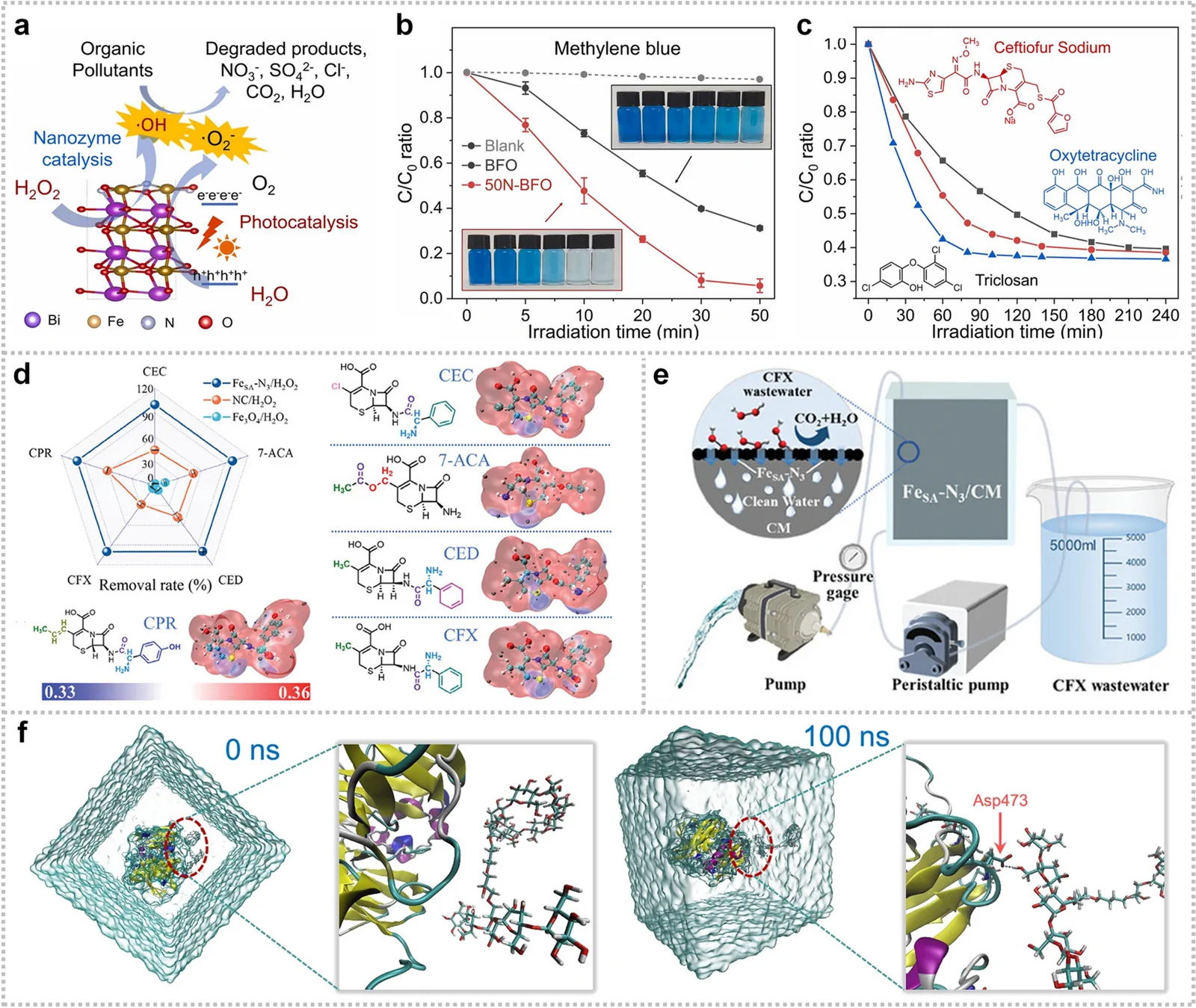
Nanozyme-mediated remediation of organic pollutants. **a** Schematic illustration of irradiation-enhanced BiFeO_3_ nanozymes for organic pollutant degradation. **b** C/C_0_ ratios for methylene blue degradation under simulated solar irradiation (1 kW m^−2^), with the inset showing corresponding photographic images. **c** C/C_0_ ratios for the degradation of cefotaxime sodium, triclosan, and oxytetracycline under simulated solar irradiation (1 kW m^−2^). Reproduced with permission [74]. Copyright 2024, Elsevier. **d** Removal efficiencies of five cephalosporin antibiotics in NC/H_2_O_2_, FeSA-N_3_/H_2_O_2_, and Fe_3_O_4_/H_2_O_2_ systems, along with their molecular structures and surface ALIE analysis. **e** Schematic of continuous oxidation of cefixime (CFX) using FeSA-N_3_/CM. Reproduced with permission [108]. Copyright 2024, Wiley–VCH. **f** MD simulations showing interactions between Cellulose-CD-MMT hydrogel and laccase at the initial state (0 ns) and equilibrium state (100 ns). The cyan box represents a 10 × 10 × 10 nm water box. Reproduced with permission [77]. Copyright 2025, Springer Nature

Organic pollutants in aquatic and environmental systems, such as PAEs used as plasticizers, and PAHs derived from food processing and combustion, pose serious ecological and health risks due to their persistence, recalcitrance, and bio-accumulative nature. To address the high ecological risks of antibiotics in aquatic environments, 16 studies to date have focused on strategies such as electronic structure modulation, oxygen-vacancy engineering, and single-atom coordination environment design to develop high-performance nanozyme systems. These approaches aim to overcome the key limitations of conventional methods, including insufficient selectivity and constrained catalytic efficiency in complex water matrices. Guo et al. modulated the electronic structure of BiFeO_3_ nanozymes via nitrogen doping (Fig. 10a) [74], significantly enhancing the electron density of Fe–N active centers and optimizing the band structure, thereby achieving photo-enhanced POD-like activity. This nanozyme increased ROS generation by 14.63-fold, achieved a 94.27% degradation efficiency for methylene blue (Fig. 10b), and degraded over 60% of multiple antibiotics (Fig. 10c), while also exhibiting magnetic recoverability, providing an efficient and feasible approach for waterborne organic pollutant remediation. To further improve the catalytic efficiency and substrate conversion rate of LAC-like nanozymes, oxygen-vacancy engineering has emerged as a core strategy. Liu et al. employed low-temperature plasma to synthesize CoNi-MOF [75] and achieved controlled oxygen-vacancy distribution by adjusting N_2_/O_2_ gas ratios, which significantly enhanced LAC-like activity and promoted O_2_ activation for ROS generation. Compared with natural laccase, this CoNi-MOF exhibited LAC-like activity with a lower Michaelis constant (*K*_*m*_ = 0.094 mM) and a higher maximum reaction rate (*V*_max_ = 31.6 × 10^–8^ M s^−1^), demonstrating superior substrate affinity and catalytic kinetics, along with high stability and low toxicity, offering a new strategy for green antibiotic remediation. For highly selective antibiotic removal, theoretical calculations have guided the design of SAzymes with notable success. Wang et al. optimized the coordination environment of FeSA-N_x_ SAzymes through theoretical modeling [108], revealing that the Fe-N_3_ configuration exhibits optimal enzyme-like activity (Fig. 10d) by lowering the free energy for •OH generation via efficient electron transfer. The FeSA-N_3_ nanozyme displayed high selectivity toward cephalosporin antibiotics (R^2^ = 0.820–0.929), and when integrated into ceramic membranes, enabled continuous and stable antibiotic removal (Fig. 10e), providing a promising strategy for the integration of nanozymes in water treatment applications. This continuous catalytic process enables efficient transformation of antibiotic pollutants under flow conditions, while facilitating their sustained removal and transport regulation within aqueous systems.

PAEs are present in polymeric materials through non-covalent interactions and can be continuously released during manufacturing, processing, and environmental aging, forming typical persistent organic pollutants. Addressing the limitations of conventional hydrolytic or oxidative treatments—namely low catalytic efficiency and poor selectivity—ten studies have demonstrated that nanozymes can construct highly efficient hydrolytic/oxidative active sites via metal-induced self-assembly or co-immobilization with natural enzymes, enabling stable and effective degradation of representative PAEs such as di(2‑ethylhexyl)phthalate (DEHP) and triphenyl phosphate (TPHP). The underlying mechanisms involve radical-mediated oxidative cleavage and multienzyme cascade catalysis. Liang et al. developed a bio-nanozyme with hydrolase-like activity by inducing histidine-enriched heptapeptides to self-assemble via zinc ions [109]. This nanozyme forms β-sheet nanofiber structures, which not only catalyze the hydrolysis of various p-nitrophenyl esters but also efficiently degrade the model plasticizer DEHP, providing a new paradigm for the design of bio-based nanozymes and their application in environmental remediation. To overcome the limited stability and reusability of natural peptide-based nanozymes in complex environments, Zhao et al. co-immobilized PtCo nanozymes and laccase on dendritic mesoporous silica nanoparticles (DMSN), forming a Lac@PtCo@DMSN composite catalytic system [76]. When the Pt:Co molar ratio was 3:1, the PtCo nanozyme exhibited optimal oxidative activity, and the composite carrier supported a high laccase loading of up to 365 mg g^−1^. Through synergistic catalysis involving •OH and O_2_^−^, this system achieved efficient PAE degradation, with an 81.83% removal efficiency within 72 h. Notably, after five reuse cycles, the degradation efficiency of dimethyl phthalate (DMP) remained 72.44%, significantly enhancing the practical applicability of nanozymes. This coupled enzymatic–nanozymatic system enables continuous pollutant transformation with stable catalytic performance under operational conditions, supporting long-term wastewater treatment applications.

PAHs characterized by strong hydrophobicity, high chemical stability, and pronounced toxicity are recognized as some of the most recalcitrant persistent organic pollutants. Their degradation efficiency is often constrained by narrow microbial substrate specificity and inhibition from metabolic intermediates. Nanozymes, by mimicking the catalytic mechanisms of natural enzymes, can facilitate oxidative ring-opening and cooperative degradation of PAHs, offering high efficiency, environmental adaptability, and controllability, thus demonstrating low-ecological risk application potential. To date, 11 studies have reported relevant findings. Das and colleagues found that *Pseudomonas fragi* DBC can efficiently degrade high molecular weight PAHs, including fluorene, pyrene, and benzo[a]pyrene [110], achieving 90%–99% removal within 28 days. The underlying mechanism involves catechol 1,2-/2,3-dioxygenases produced by the strain, which exhibit high affinity toward benzo[a]pyrene and its intermediates. qRT-PCR analysis confirmed significant upregulation of the dioxygenase genes, highlighting the critical role of enzyme-mediated substrate specificity in regulating microbial degradation kinetics. However, microbial degradation approaches are limited by slow reaction rates, poor stability, and low controllability, restricting their practical efficacy in water treatment. Zhang et al. developed a cellulose-based hydrogel doped with β-cyclodextrin (CD) and montmorillonite nanosheets (MMT) (cellulose-CD-MMT) [77]. CD provides molecular recognition for capturing hydrophobic pollutants such as PAHs, while MMT significantly enhances the mechanical performance of the hydrogel. Using a scalable enzyme immobilization strategy, laccase was efficiently assembled onto the hydrogel carrier. Molecular dynamics (MD) simulations (Fig. 10f) revealed that the –OH groups of the hydrogel form strong covalent-anchored hydrogen bonds (CAHB) with the –COOH group of Asp473 in laccase, contributing most to hydrogen bonding and markedly enhancing enzyme stability and activity. This approach successfully achieved efficient degradation of PAHs, antibiotics, and per- and polyfluoroalkyl substances (PFAS) in real wastewater, overcoming the limitations of conventional enzyme immobilization techniques and providing a new avenue for sustainable aquatic pollutant remediation and next-generation programmable enzyme technologies.

Current studies indicate that nanozymes, through electronic structure modulation, oxygen vacancy engineering, and precise design of single-atom active sites, can establish catalytic systems that combine high efficiency, selectivity, stability, and recyclability, providing a core technological foundation for the degradation of representative organic pollutants such as antibiotics, PAEs, and PAHs. Integration with multifunctional carrier modifications or enzyme immobilization strategies can further enhance nanozyme performance in complex environments. Nevertheless, existing nanozyme systems still face critical challenges in real-world water matrices, including narrow substrate adaptability, rapid decay of catalytic activity, limited long-term recyclability, and high costs for scalable production. Future efforts should focus on expanding substrate spectra, optimizing multifunctional composite carriers to enhance environmental adaptability and cyclic stability, and developing low-cost, scalable fabrication methods, alongside systematic assessment of ecological safety, thereby providing practical and sustainable strategies for efficient water treatment of organic pollutants.

#### Pesticide Pollutants

In recent years, nanozymes have achieved breakthrough progress in addressing OPs pollution, particularly demonstrating high efficacy in the targeted hydrolytic cleavage of P-O bonds. To date, 27 studies have established green and efficient nanozyme-based approaches for OPs remediation, providing a solid foundation for the development of environmentally friendly pollutant treatment technologies.

**Fig. 11:**
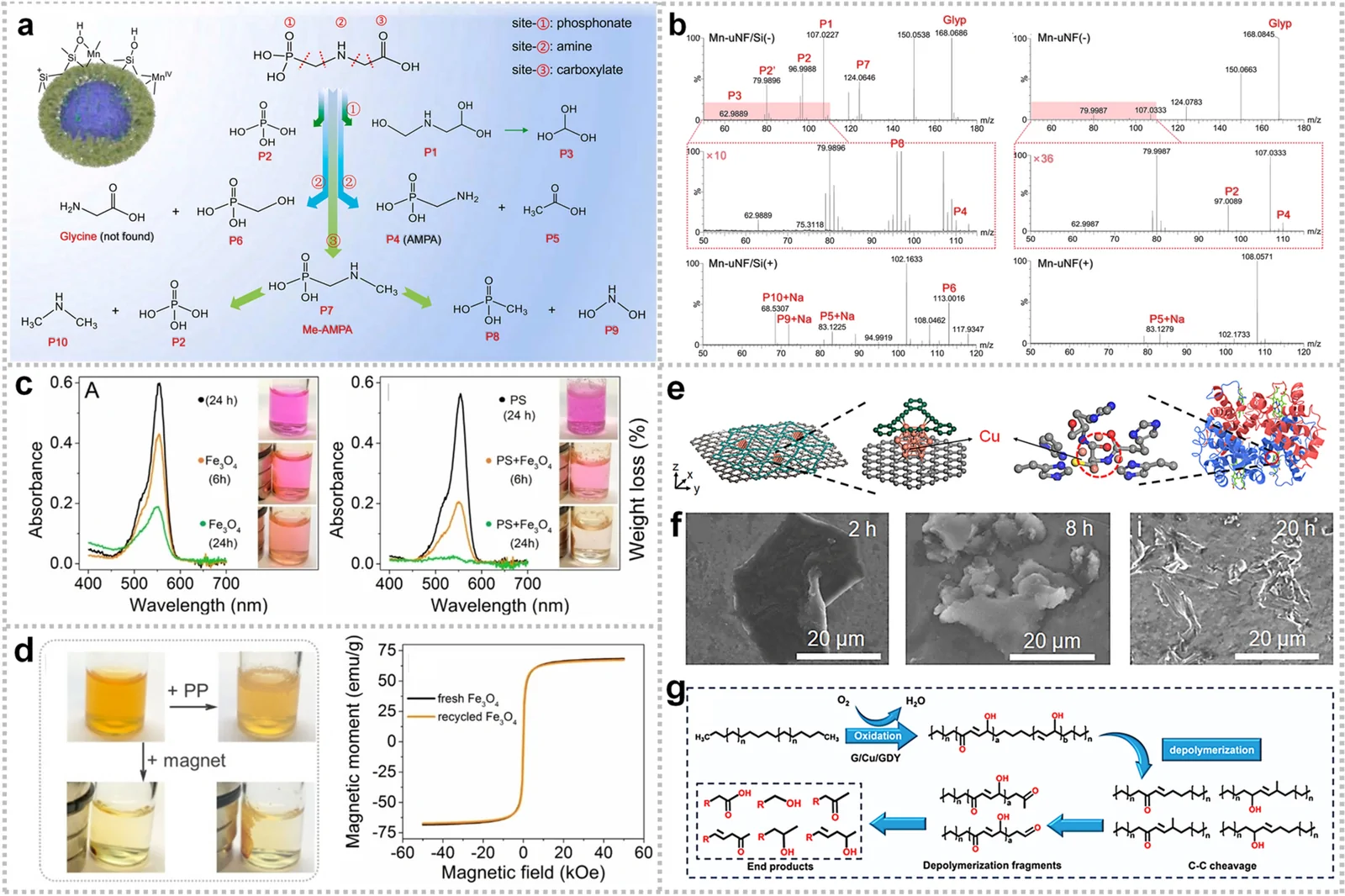
Nanozyme-mediated degradation of Pesticide Pollutants and microplastics.** a** Reaction pathway for glyphosate (Glyp) degradation catalyzed by Mn-uNF/Si and Mn-uNF. **b** Mass spectrometric analysis of Glyp degradation catalyzed by Mn-uNF/Si and Mn-uNF. Reproduced with permission [78]. Copyright 2024, Elsevier. **c** UV–vis spectra and photographs for the degradation and decolorization of 10 µM RhB using 60 µg mL^−1^ Fe_3_O_4_. **d** Magnetic separation of microplastics using recovered Fe_3_O_4_ after degradation experiments. Reproduced with permission [81]. Copyright 2022, Wiley–VCH. **e** Schematic illustration of the G/Cu/GDY nanozyme structure. **f** SEM images of microplastics at different reaction times: 2 h, 8 h, and 20 h. **g** Degradation pathway of LDPE catalyzed by G/Cu/GDY. Reproduced with permission [83]. Copyright 2025, Wiley–VCH

The toxicity of OPs primarily originates from the P-O bond of phosphodiester within their molecular structure, making the construction of catalytic systems capable of efficient P-O bond cleavage a critical objective in OPs remediation. Nanozymes, by mimicking the catalytic activity of natural phosphoesterases, can achieve specific hydrolytic cleavage of P-O bonds. Wu and colleagues developed a Mn uNF/Si nanozyme by in situ growth of ultrathin Mn-MOF nanofibers on silica spheres, exhibiting high biomimetic phosphotriesterase-like activity (Fig. 11a) [78]. This nanozyme combines Lewis–Brønsted acid–base dual catalysis, wherein Mn(II) acts as a Lewis acid to activate the P-O bond, and the Brønsted base site promotes nucleophilic attack by water molecules, enabling efficient dephosphorylation of OPs. Mass spectrometry analysis revealed that glycine preferentially cleaves the C-N bond under the influence of phosphate and carboxylate functional groups to form aminomethyl phosphonic acid (AMPA). Subsequently, *N*-methyl-AMPA (Me-AMPA) undergoes further degradation, with C-P bond cleavage yielding P2. The accumulation of P2 is primarily associated with the initial C-P bond cleavage, revealing the time-dependent mechanism of Mn-uNF/Si nanozyme-mediated OPs degradation (Fig. 11b). Building upon this, Cao et al. constructed an aptamer-modified Zr-MOF nanozyme (UiO-66-APT) to achieve the specific hydrolysis of paraoxon (PXN) [111]. The study demonstrated that the binding mode of the aptamer on the nanozyme surface regulates the interaction between substrates and enzyme-like active sites, thereby influencing catalytic activity and substrate selectivity. This nanozyme system efficiently hydrolyzed phosphatase substrate (PNPP) and PXN. Moreover, specific molecular recognition of PXN by the aptamer increased the local substrate concentration near the active sites, further enhancing catalytic efficiency and enabling selective regulation. Sun et al. proposed a ligand-engineered Zr-MOF nanozyme to alleviate phosphorus deficiency in plants [112]. By introducing halogen substituents at the ortho position of the terephthalate ligand, the Lewis acidity and defect structure of Zr nodes were modulated, enhancing phosphatase-like activity and promoting organophosphate mineralization. The mechanism involves strengthened substrate adsorption and P-O bond activation, which reshapes plant metabolism and significantly improves growth under low-phosphorus conditions, demonstrating the potential of Zr-MOF nanozymes in environmental remediation and precision agricultural nutrient management. Integration of membrane separation with nanozyme catalysis offers a promising approach for waterborne OPs removal. Zhang et al*.* synthesized UiO-66(Ce) nanozymes via a hydrothermal method and immobilized them on poly (vinylidene fluoride) (PVDF) membranes to construct a UiO-66(Ce)/PVDF composite catalytic system[79]. This system efficiently hydrolyzed the phosphatase substrate p-nitrophenyl phosphate (p-NPP). Potentiometric acid–base titration was employed to characterize different proton species in the Ce-MOF, leading to the proposal of an OH⁻-mediated Ce(III)/Ce(IV)-OH dual-site cascade catalytic mechanism, in which Ce(IV)-OH activates the P-O bond, while Ce(III)-OH provides nucleophilic attack sites. Integration of this composite membrane into a rotor-type aeration device successfully achieved efficient removal of OPs in real Dianchi Lake water samples, providing a novel design strategy and mechanistic insight for complex waterborne OPs remediation. To further develop highly efficient and sustainable catalytic materials for OPs degradation, Liao et al. constructed a multifunctional fluorescent nanozyme (Eu@Ce/UiO-67) through hydrothermal synthesis and post-synthetic modification [80], which efficiently catalyzes paraoxon degradation. Comparative analysis of activation energies for paraoxon hydrolysis by UiO-67 and Eu@Ce/UiO-67 and mechanistic studies of Eu@Ce/UiO-67 during paraoxon hydrolysis revealed that Ce-O-Zr clusters exhibit lower energy barriers. Ce^4+^ doping significantly increases structural defect sites, further enhancing phosphatase-like activity and substantially improving paraoxon degradation efficiency.

Nanozymes can efficiently activate P-O bonds and convert toxic OPs into harmless phosphate species by mimicking the metal catalytic centers of natural phosphoesterases, such as the Zn-Zn bimetallic center mimicked by Zr-MOFs and their hydrolytic mechanisms. Ce-MOF composite membrane systems further achieve substrate enrichment and efficient hydrolysis through dual-site cooperative catalysis, providing an engineered solution for waterborne OPs remediation. However, current nanozyme systems still face critical challenges, including activity attenuation in complex aqueous matrices, limited substrate specificity, and insufficient stability and recyclability. Future research should focus on defect engineering, heteroatom doping, and hydrophilic–hydrophobic interface optimization to enhance catalytic activity, selectivity, and environmental adaptability. Concurrently, the development of recoverable, broad-spectrum nanozyme materials will be essential to advance the scalable and sustainable application of OPs remediation technologies.

#### Microplastics

Nanozymes can mimic natural hydrolases and oxidases or induce the generation of reactive species via surface engineering, enabling efficient and selective cleavage of C–C bonds within polymer chains. To date, 16 core studies have demonstrated that strategies such as interfacial catalysis enhancement, precise electronic transfer modulation, and targeted generation of reactive species significantly improve polymer chain scission efficiency and deep mineralization, while optimizing catalytic selectivity, environmental adaptability, and reaction controllability. Table 3 summarizes the conditions for CO_2_ release during different microplastic degradation processes. These advances highlight the high-efficiency, tunable, and sustainable potential of nanozymes for microplastic degradation.

**Table 3:** CO_2_ release conditions for different processes for degrading MPs

Treatment	Temperature	CO_2_ emissions (kg/kg MPs)	References
Nanozyme	100 °C	1.53	[113]
Incineration	> 800 °C	3.01	[114]
Biodegradation	Normal temperature, long time	0.65–5.28	[115]
Enzymatic-electrochemical degradation	Normal temperature	4.81	[116]

Although natural enzymes exhibit high catalytic specificity, their application in deep microplastic degradation and mineralization is severely limited by susceptibility to inactivation in complex environments, low polymer chain cleavage efficiency, and poor reusability. To address these limitations, Liu et al. developed a hydrophilic-exposed Fe_3_O_4_ nanozyme [81], which leverages surface hydrogen bonding to achieve efficient adsorption of diverse microplastics while maintaining excellent peroxidase-like activity (Fig. 11c). Under reaction conditions approaching the plastic’s melting temperature, this nanozyme achieved nearly 100% degradation efficiency and, owing to its magnetic properties, enabled facile recovery and reuse (Fig. 11d). Despite the promising performance of Fe_3_O_4_ nanoparticles, their catalytic activity remains constrained by the number of surface-active sites and limited electronic regulation capability. SAzymes, which maximize atomic utilization, offer a new route to overcome this bottleneck. Gao et al. constructed a copper single-atom graphitic carbon nitride nanozyme (Cu SAs) [82], achieving over 90% mineralization of polystyrene into CO_2_, water, and short-chain carboxylic acids without generating toxic intermediates, thus eliminating secondary pollution risks associated with conventional degradation methods. The high catalytic efficiency of natural enzymes depends on precise electronic interactions between the active center and cofactors, whereas conventional SAzymes cannot fully mimic this 3D electronic architecture, limiting their activity. Inspired by the catalytic mechanism of natural laccase, Sun et al. proposed a graphdiyne-assisted strategy for constructing a 3D biomimetic nanozyme (G/Cu/GDY) (Fig. 11e) [83]. This system employs multiple copper-cluster active centers to efficiently activate O_2_ and generate ROS, enabling rapid oxidation and chain scission of low-density polyethylene (LDPE). Optimal performance was observed at pH 6, with substantial molecular weight reduction within 20 h, converting high molecular weight polymers into low molecular weight fragments. SEM images (Fig. 11f) reveal progressive fragmentation of LDPE films into fine fibers over 20 h, indicating a degradation process involving oxidation-induced chain segment activation, C–C backbone cleavage, and subsequent deep mineralization (Fig. 11g). This 3D G/Cu/GDY nanozyme, constructed via large d-π orbital hybridization, not only provides a generalizable structural design strategy but also offers critical insights for the rational design of nanozymes targeting microplastic degradation. However, most of the above studies remain at the laboratory scale, with limited validation in real ecological environments, and their long-term stability, catalytic efficiency, and ecological safety in complex environmental systems still require further evaluation. To bridge this gap, Yao et al. developed a multifunctional copper-doped polydopamine-functionalized magnetic silica adsorbent (Fe_3_O_4_@SiO_2_@CP) capable of rapid capture [117], multiple-cycle reuse, and label-free on-site detection of nano- to microscale plastic particles. By integrating laccase-mimetic catalytic activity with machine learning, the platform enables highly selective identification of plastic types and concentrations and has been validated for efficient capture and detection of low-concentration micro- and nanoplastics in both natural water sources and everyday water. This approach provides a practical strategy for the resource recovery of micro- and nanoplastics and their ecological remediation. Beyond capture and detection, catalytic conversion of microplastics into value-added products is crucial for sustainable resource utilization. Zhang et al. utilized the peroxidase-like activity of Fe_3_O_4_ nanozymes to generate •OH under mild conditions (100 °C) [113], enabling selective C–C bond cleavage in microplastics. Within 7 h, approximately 50% of polyethylene was converted into low-toxicity, high-value products, some of which could serve as microbial carbon sources. Life cycle assessment indicated a carbon footprint of 1.53 kg CO_2_ eq/kg MPs, with a net gain of about 0.73 USD per kilogram of microplastics processed. This approach provides a feasible strategy for low-carbon valorization of microplastics and promotes ecological cycling in agricultural systems.

Current studies demonstrate that nanozyme technology can significantly enhance the degradation efficiency, catalytic stability, and reusability of microplastics and PET. However, challenges remain for industrial-scale application, including insufficient stability under complex operational conditions, reliance on elevated temperatures in most systems, and the lack of systematic evaluation of long-term cycling performance. Future efforts may focus on optimizing nanozymes through multi-metal cooperative active sites, composite carriers, and low-energy auxiliary strategies to achieve efficient circular degradation of plastics and recovery of valuable degradation products.

## Conclusions and Outlook

Eco-nanozymology, an emerging interdisciplinary research paradigm integrating nanoscience, enzymology, and ecological principles, is fundamentally reshaping our understanding of energy conversion and environmental remediation. This review systematically delineates the principles of eco-nanozymes in key biogeochemical cycles, including carbon, nitrogen, and hydrogen, as well as in energy conversion and environmental remediation processes. In energy conversion, eco-nanozymes enable highly efficient artificial nitrogen fixation, carbon fixation, methane oxidation, and hydrogen production, while markedly improve the performance of secondary batteries and EBFCs. These advances provide novel material strategies and design paradigms for constructing environmentally-friendly, cost-effective, and sustainable energy strategies and devices. In environmental remediation, eco-nanozymes achieve efficient degradation and resource-oriented valorization of low-value biomass, including lignin, agricultural residues, livestock manure, and microplastics. These strategies substantially enhance reaction rates, substrate selectivity, and catalytic stability, driving pollution treatment toward green, closed-loop, and sustainable resource recycling. Collectively, the development of eco-nanozymology offers a systematic framework to address global challenges in energy scarcity, environmental pollution, and climate change, while establishing a robust theoretical and technological foundation for the advancement of sustainable catalysis.

**Fig. 12:**
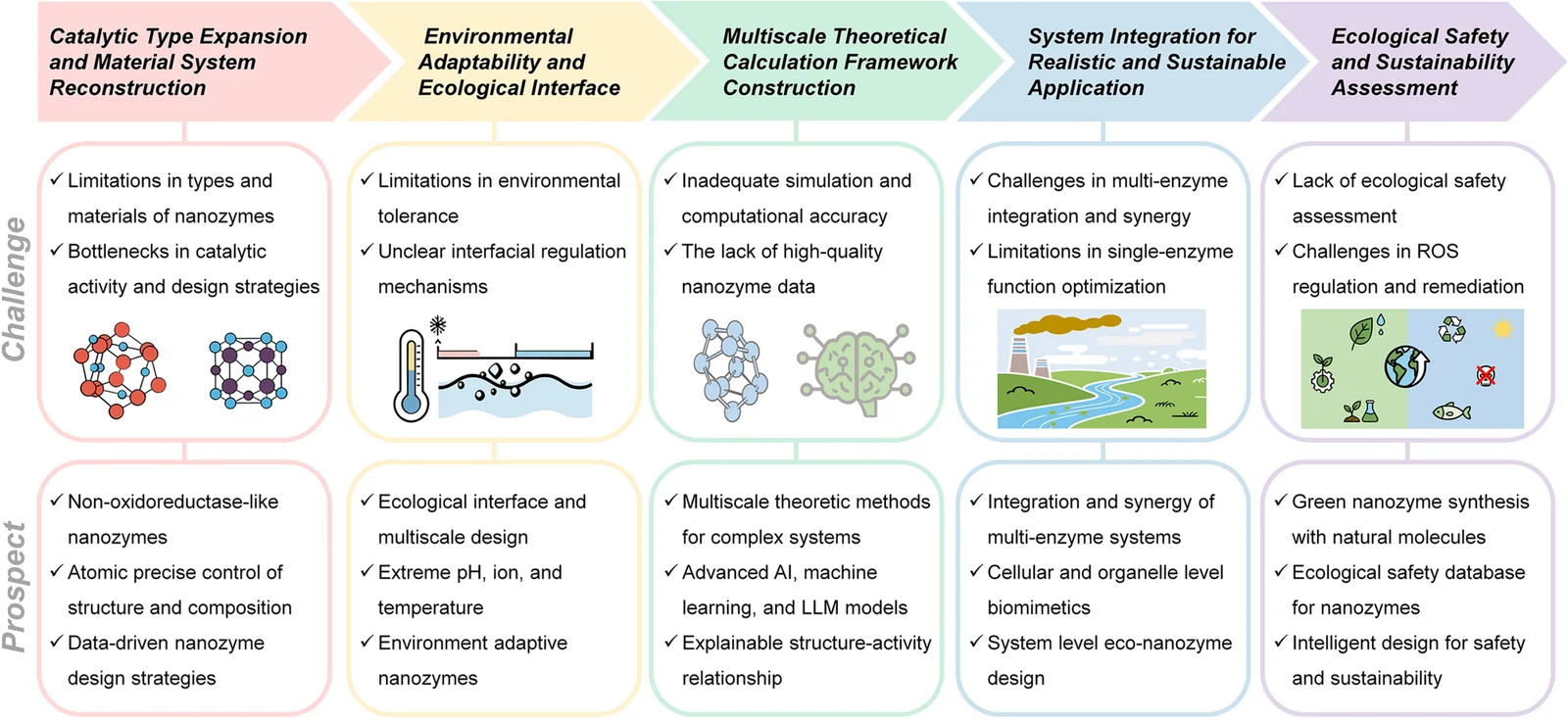
Future prospects for eco-nanozymology

Looking ahead, eco-nanozymology presents both challenges and opportunities, which can be further advanced along five key directions: (1) expansion of catalytic types and reconstruction of material systems; (2) improvement in environmental adaptability; (3) construction of multiscale theoretical calculation frameworks; (4) integration of eco-nanozyme systems for realistic and sustainable applications; (5) establishment of ecological safety and sustainability assessment framework (Fig. 12).

Building on this foundation, the integration of emerging technologies such as AI-driven design, multi-scale modeling, high-throughput synthesis and microenvironmental control can further enable precise optimization of nanozyme catalytic activity, selectivity and stability. At the same time, quantifying the environmental impacts of materials through lifecycle and carbon footprint analyses can guide low-carbon, green synthesis and the circular utilization of resources. The establishment of comprehensive policy and regulatory frameworks including ecological risk assessment, green manufacturing standards, and long-term monitoring will ensure the safe application of eco-compatible nanozymes. The development roadmap can be implemented in stages. In the short term (1–3 years), focus on performance evaluation and ecological risk quantification. In the medium term (3–7 years), achieve AI-guided optimization and system integration. In the long term (7–15 years), realize low-carbon, large-scale production and broad application across energy, environmental remediation, and ecosystem management, thereby providing technological and policy support for sustainable development.

### Expansion of Catalytic Types and Reconstruction of Material Systems

Since the discovery of nanozymes by Yan and colleagues in 2007 [118], the field has experienced rapid growth. Over 420 research groups worldwide have engaged in nanozyme studies, developing thousands of nanozymes encompassing six major catalytic types, involving oxidoreductases, hydrolases, lyases, isomerases, transferases, and ligases. The application scope of nanozymes has gradually expanded from biochemical analysis to medical therapy, green synthesis, environmental remediation, and renewable energy. In contrast, the development of eco-nanozymes has lagged behind. In terms of catalytic types, eco-nanozymes are primarily concentrated in oxidoreductase- and hydrolase-like nanozymes. Although the oxidoreductase-like nanozymes have formed relatively well-established systems, hydrolase-like nanozymes face challenges such as limited catalytic types, singular material systems, and narrow developmental pathways [119]. Regarding catalytic activity, despite notable progress in eco-nanozyme, their overall catalytic activity generally remains lower than that of natural enzymes, with several common underlying reasons. For instance, the precise control of active site structures is difficult, electron transfer kinetics are constrained, and materials often exhibit insufficient adaptability to complex and dynamic reaction environments. Many reactions still occur on non-specific surface sites, rendering the structure–activity relationship of active sites unclear. Moreover, existing design strategies predominantly rely on single-component inorganic materials, lacking effective mimicry of the hierarchical structures, synergistic effects, and organic/biological components of natural enzymes. More importantly, current development approaches are largely empirical and trial and error based, with limited theoretical guidance or ML assistance, thereby restricting further enhancement of substrate specificity, catalytic activity, and material designability [120].

In recent years, breakthroughs in nanotechnology, computational modeling, and advanced characterization techniques have opened new avenues for expanding nanozyme catalytic types and reconstructing their material systems. Through biomimicking the geometric and electronic structures of natural enzymes, the construction of hierarchical nanostructures and electric field distributions that possess the characteristic features of natural enzymes not only increases the effective active surface area but also optimizes mass transport and interfacial dynamics during reactions [83, 121–123]. By integrating diverse catalytic types and co-constructing organic/biological functional units with inorganic matrices, the adaptability, selectivity, and stability of nanozymes in complex ecological environments can be substantially enhanced [124–128]. Furthermore, the incorporation of smart-responsive functionalities endows nanozymes with dynamic regulatory capabilities akin to allosteric modulation in natural enzymes, significantly improving their environmental adaptability and catalytic tunability [129, 130]. Concurrently, ML is progressively emerging as a core technology driving the rational design of nanozymes [131–133]. By deepening the mechanistic understanding of nanozyme catalysis and precisely elucidating the high-dimensional relationships between physical descriptors and catalytic activity, ML can not only identify potential materials beyond the conventional design space but also markedly shorten development cycles, propelling nanozymes into a data-driven accelerated development era [134].

### Improvement in Environmental Adaptability

Enzymes can significantly accelerate a wide range of biochemical reactions under mild physiological or near-physiological conditions and play a central role in complex molecular transformations across industrial processing, biomedical applications, and life science. However, under harsh pH conditions, e.g., strong acids or bases, the primary structure of enzymes and their finely tuned higher-order conformations are easily disrupted, leading to subunit dissociation and conformational denaturation, which ultimately results in a substantial loss or complete abrogation of catalytic activity. In contrast, the active centers of nanozymes are typically exposed on the material surface and are constituted by specific atomic coordination structures, surface defects, or preferential crystal facets. This open configuration significantly enhances substrate accessibility and interfacial reaction kinetics, enabling nanozymes to maintain robust activity even under extreme pH, temperature, or redox conditions. Moreover, the active sites of nanozymes are modular or relatively independently distributed, so local structural perturbations have limited impact on overall catalytic activity, conferring high stability and environmental tolerance distinct from natural enzymes. On this basis, recent studies have further explored the construction of programmable local microenvironments to enhance the reaction selectivity and catalytic activity of eco-nanozymes, with a particular focus on mimicking natural enzymes the fine regulation of acid–base balance, proton transfer, and intermediate stabilization around active centers of natural enzymes. For example, Wei et al. employed a MOF as a nanozyme model [28], in which low molecular weight poly (acrylic acid) (PAA) was introduced as a Brønsted acid and spatially confined within the internal pores of a MOF-based POD-like nanozyme. The provision of protons by PAA creates an acidic microenvironment, allowing the MOF nanozyme to achieve optimal catalytic activity even under neutral conditions.

In natural ecosystems, salinization represents a typical abiotic stress that often leads to excessive accumulation of ROS in crops, causing nucleic acid damage, membrane lipid peroxidation, and Na^+^/K^+^ imbalance, which can exceed the clearance capacity of SOD. To address this challenge, eco-nanozymes have been designed to mimic natural enzymatic catalytic mechanisms or to construct artificial microenvironments, thereby achieving adaptive regulation under interfacial conditions [135]. Under saline–alkaline or neutral environments, POD-like nanozymes can maintain catalytic activity by generating localized acid–base microenvironments, enhancing electron transfer efficiency, or simulating Mg^2+^ active sites. This, in turn, strengthens crop stress resistance, restores redox homeostasis, and mitigates the adverse effects of ion imbalance on photosynthesis and yield. Furthermore, SOD-like nanozymes can directly scavenge superoxide radicals, functionally mimicking the Mg^2+^-pH synergistic mechanism of RuBisCO light activation, thereby further enhancing plant salt tolerance.

Beyond pH and ionic adaptability, temperature tolerance represents another critical aspect in the study of eco-nanozymes. Psychrophilic enzymes, which efficiently catalyze reactions at low temperatures (0 °C or below), have broad applications in food, pharmaceutical, and environmental processes. However, their poor thermal stability limits practical utility. By mimicking the flexible conformations of psychrophilic enzymes and modulating structural factors such as metal oxidation states and oxygen vacancies, nanozymes have been successfully engineered to achieve high catalytic efficiency under low-temperature conditions. For instance, ε-MnO_2_-based nanozymes exhibit robust activity and stability in cold environments, highlighting the potential of nanozymes for low-temperature catalysis and biomass degradation, and providing new material platforms and technological pathways [104, 136, 137].

Looking forward, the advancement of eco-nanozymes necessitates further development constrained by ecological processes, progressing toward multiscale biomimetic design [27], with a focus on constructing interfacial catalytic systems capable of self-adapting to pH gradients, ionic strength, and organic matter composition. On the one hand, mechanisms such as the Mg^2+^-pH coupled activation of RuBisCO under light-driven conditions can inspire the development of interfacial modulation strategies that integrate photonic, ionic, and protonic cues, enabling nanozymes to maintain high catalytic efficiency in neutral, saline, and alkaline environments. On the other hand, by leveraging high-spin state regulation, oxygen vacancy engineering, and Jahn–Teller effects to introduce conformationally equivalent flexibility, catalytic centers can retain activity even under low-temperature conditions. Notably, studies on psychrophilic nanozymes indicate that low-temperature catalysis requires consideration of non-classical kinetics such as quantum tunneling. Therefore, future eco-nanozyme design should integrate interfacial electronic structure modulation with quantum tunneling effects to establish systems capable of efficient ecological catalysis under extreme environmental conditions.

### Construction of Multiscale Theoretical Calculation Frameworks

Natural enzymes, owing to their multifunctionality, high selectivity, and exceptional catalytic activity, serve as critical benchmarks in biocatalysis research [138]. However, achieving comparable performance with computationally designed nanozymes in non-natural reactions remains highly challenging. Despite advances in deep learning, quantum chemical calculations, and multiscale dynamic simulations, current methodologies still fall short in fully and accurately capturing real catalytic processes. Structure prediction and MD simulations rely on approximate potential energy surfaces, making it difficult to resolve key dynamic phenomena such as highly coordinated conformational rearrangements, transition state stabilization, and long-range allosteric regulation within catalytic cycles. Processes involving metal centers, cofactors, and PCET entail complex electronic correlations, rendering detailed elucidation of active site electronic structures and reaction pathways particularly challenging. Furthermore, the scarcity of high-quality kinetic and structural data constrains the reliable training and generalization of structure–activity relationship models [139]. Collectively, these factors limit the ability of computationally designed nanozymes to match the microenvironmental control and catalytic efficiency exhibited by natural enzymes.

To overcome these bottlenecks, current nanozyme research is progressively establishing a synergistic paradigm that integrates computational modeling, ML, and experimental validation. By combining density functional theory (DFT) calculations, ML algorithms, and experimental database construction, researchers can systematically quantify the relationships between material structural features, active site properties, and catalytic activity, enabling precise prediction and targeted optimization of nanozyme activity, substrate selectivity, and reaction mechanisms. On the one hand, activity descriptors derived from DFT [140], coupled with ML-based high-throughput screening and performance prediction models, have significantly enhanced the accuracy and efficiency of material design. On the other hand, in-depth elucidation of natural enzyme structural mechanisms allows for the biomimetic construction of catalytic interfaces in nanomaterials with controllable microenvironments and hierarchical synergistic effects, simultaneously improving catalytic activity and substrate selectivity [141]. Furthermore, establishing a cross-scale computational framework facilitates systematic correlation of electronic structures, reaction kinetics, and macroscopic performance, providing robust theoretical support for designing efficient nanozyme systems under complex application scenarios. By integrating multiple methodologies to construct a quantitative structure–activity relationship framework, these studies not only furnish a theoretical basis for the coupling of structure, function, and dynamics in eco-nanozymes but also offer new technical avenues for optimizing environmental adaptability and substrate specificity.

Future efforts urgently require the development of a multiscale theoretical calculation framework to systematically elucidate the complex coupled mechanisms governing eco-nanozymology. Such a framework should establish cross-scale linkages spanning from electronic structures and interfacial reaction kinetics, to mesoscale mass transport behaviors, and further to ecosystem-level functional feedback processes, thereby enabling the construction of an integrated theoretical model capable of simultaneously predicting the catalytic performance and ecological consequences of nanozymes. Through the deep integration of density functional theory (DFT) calculations, reaction network simulations, and machine learning (ML) approaches, key catalytic parameters can be systematically analyzed, including adsorption energies, reaction barriers, and electron transfer pathways at active sites, which represent critical microscopic kinetic features. On this basis, these insights can be incorporated into reaction network models describing representative ecological transformations, such as reactive oxygen species (ROS) conversion and carbon and nitrogen cycling reactions, thereby enabling cross-scale coupling between microscopic catalytic processes, mesoscale transport phenomena, and macroscopic ecological responses. Within this theoretical framework, ML-driven data analysis can further establish quantitative structure–activity relationships between structural descriptors (e.g., coordination environments, defect structures, and electronic features), microenvironmental regulatory factors (such as pH, ionic strength, and substrate concentration), and macroscopic catalytic performance. This process will facilitate the extraction of platform-transcending physical descriptors and generalizable design principles. Building upon these advances, the development of broadly applicable theoretical models and computational frameworks will provide essential foundations for achieving predictable design, controllable regulation, and functional optimization of eco-nanozymes in complex ecosystems, ultimately advancing the field toward a stage characterized by mechanistic interpretability, predictive capability, and practical implement ability.

### Integration of Eco-nanozyme Systems for Realistic and Sustainable Applications

In nature, enzymes operate within highly orchestrated division of labor systems and cascade catalytic networks, driving the vast and complex biochemical reactions of living organisms. While individual enzymes typically catalyze a single reaction for a specific substrate, in real cellular environments, enzymes rarely function in isolation. Instead, they achieve functional integration through cascade reactions, coupled metabolic pathways, and multienzyme complexes. For instance, natural photosynthetic systems efficiently convert water and CO_2_ into energy-rich carbohydrates via light-harvesting complexes (LHCs), enzymes, and various cofactors e.g., coenzyme Q, adenosine triphosphate (ATP), and nicotinamide adenine dinucleotide phosphate (NADPH). This highly specialized and tightly coupled multifunctional unit exemplifies why constructing artificial photosynthesis not only requires material performance optimization but also poses critical challenges in functional unit coordination and system integration. In contrast, current nanozyme research predominantly focuses on the development and optimization of single enzyme-like functions, lacking hierarchical integration and efficient cooperation across multienzyme systems, which limits their ability to mimic natural enzyme networks in complex biological processes or engineered systems.

In recent years, nanozymes have demonstrated considerable potential for constructing systems that mimic natural multienzyme cascade systems. By precisely tuning the structure and functionality of nanozymes and employing biomimetic assembly strategies, it is increasingly possible to achieve spatiotemporal coordination and functional coupling among multiple catalytic units, thereby advancing nanozyme systems from single-enzyme biomimicry toward multilevel cascade biomimicry. For example, Han et al. mimicked chloroplast functions to successfully construct artificial photosynthetic cells capable of light-driven carbon fixation [142]. In this system, ATP synthase, photosystem II, and phycocyanin were recombined within phospholipid vesicle membranes to create photosynthetic organelles, which were then coupled with a multienzyme carbon fixation cascade system. Under illumination, α-ketoglutarate was converted into acetyl-CoA and oxaloacetate, achieving efficient light-driven carbon fixation and laying the foundation for the development of self-powered artificial cells.

In the future, the development of eco-nanozymes is expected to shift from a materials design paradigm centered on single-enzyme activities toward system-level biomimetic construction that addresses real ecological processes. Through synergistic regulation of multiple active sites, spatiotemporal coupling of cascade reactions, and hierarchical design of biomimetic compartmentalized structures, eco-nanozymes hold the potential to reconstruct energy conversion and environmental remediation networks within complex ecological environments, encompassing ROS regulation, organic pollutant degradation, and key biogeochemical processes such as carbon and nitrogen cycling, thereby significantly enhancing overall catalytic efficiency, selectivity, and operational stability. Simultaneously, the synergistic application of multiscale theoretical calculation frameworks, in situ and multidimensional characterization techniques (e.g., operando spectroscopy, in situ electron microscopy, and isotopic tracing), and data-driven strategies will provide essential support for the rational structural design, performance prediction, and long-term ecological behavior assessment of eco-nanozymes, advancing them toward sustainable, tunable, and scalable practical applications. This approach not only enables cross-scale coupling between microscopic catalytic mechanisms and macroscopic ecological effects but also establishes a theoretical and methodological foundation for the predictable design and functional optimization of eco-nanozymes.

### Establishment of Ecological Safety and Sustainability Assessment Framework

Establishing a comprehensive ecological safety and sustainability assessment framework for nanozymes is of central importance for systematically elucidating their environmental behavior, toxicological effects, and long-term stability, thereby ensuring their safe and sustainable deployment in practical applications. Although eco-nanozymes have demonstrated remarkable advantages in energy conversion and environmental remediation, their ecological safety and sustainability remain insufficiently evaluated. Catalytic behaviors that appear highly efficient under laboratory or engineered conditions may undergo physicochemical alterations upon exposure to real environmental media, such as air, soil, or water, potentially giving rise to ecological risks. In particular, the dissolution, adsorption, or structural reconstruction of metal-based nanoparticles in environmental media can lead to dynamic changes in stability, mobility, and bioavailability, triggering novel toxic effects. Moreover, the capacity of eco-nanozymes to modulate ROS is closely linked to their ecological safety, because ROS is key reactive species in the environment that influence carbon and nitrogen cycling, pollutant transformation, and microbial community dynamics. However, their heterogeneous distribution across micro-interfaces, such as the rhizosphere or charosphere, and the mechanisms governing their dynamic generation remain poorly understood, limiting the potential for soil carbon sequestration and bioremediation [143]. By precisely regulating ROS generation and clearance, eco-nanozymes can stabilize local redox states, playing a pivotal role in ecological restoration. Therefore, the development of green nanozymes based on natural, non-toxic materials represents a critical direction for minimizing ecological risks.

In recent years, a variety of natural biomacromolecules, such as peptides, proteins, and plant extracts, have been employed to fabricate metal-hybrid nanostructures, attracting widespread attention due to their low cost, abundant availability, and facile preparation. For instance, extracts from *Aesculus* leaves, *Astragalus* gum, silk fibroin, garlic, and quercetin have all been successfully utilized for the green synthesis of nanozymes [144]. However, natural enzymes rely on precisely arranged amino acid residues and cofactors to maintain high catalytic activity, and their structures are prone to destabilization under environmental perturbations, limiting their long-term applicability in complex settings. To address this challenge, supramolecular self-assembly offers an effective strategy to construct cofactor-dependent nanozymes by regulating amino acid arrangement [145]. Moreover, peptide molecules can assemble via weak interactions into peptide-based nanozymes with enzyme-like activity, demonstrating broad potential in chemical catalysis and biomedicine [3, 146]. Nevertheless, dynamic control over their catalytic activity and selectivity remains challenging. Qi and colleagues achieved dynamic modulation of catalytic activity through reversible phosphorylation/dephosphorylation modifications [147]. Cui and co-workers, on the other hand, used chiral control to promote the formation of stable folded structures during metal–peptide assembly, thereby markedly enhancing the biostability of peptide-based nanozymes [148].

Future research should prioritize the establishment of a comprehensive eco-nanozyme safety database, deeply integrated with artificial intelligence (AI) predictive models. This database should systematically compile multidimensional information, including composition, particle size and morphology, surface chemical functionalities, enzyme-like activities (e.g., redox capacity, ROS generation rate), environmental mobility, degradation products, and ecological toxicity metrics, enabling high-throughput identification and quantitative assessment of ecological risks. Modeling approaches based on random forests, graph neural networks, and explainable AI (XAI) can predict toxicity endpoints (e.g., EC50, NOEC), environmental fate, and long-term ecological impacts under specific structural and environmental conditions, while identifying key toxicity-driving factors. Such insights provide mechanistic guidance for the rational design of low-risk, high-stability, and environmentally safe nanozymes, for instance, through particle size control, surface functionalization, encapsulation strategies, and tunable degradation rates to minimize ecological hazards. Overall, the future development of eco-nanozymes will increasingly emphasize ecological safety and sustainability assessment, while facilitating their practical deployment in energy conversion and environmental remediation. Achieving this requires a systematic evaluation of nanozyme stability, mobility, and bioavailability across diverse environmental matrices, such as aquatic systems, soils, and sediments, coupled with rational design strategies that balance catalytic performance with minimal ecological risk. By integrating data-driven predictive modeling with experimental validation, the safe, intelligent, and sustainable design of nanozymes can be accelerated, ensuring their long-term stability and functionality in complex ecological environments, thereby providing reliable and durable technological support for pollution mitigation and clean energy development.

## Appendix 1

To improve readability and ensure consistency of terminology, the key concepts and abbreviations used in this review are defined as follows:

- *Enzyme-like activity* Refers to the catalytic functions exhibited by nanomaterials under mild conditions that mimic natural enzymes, including oxidation, reduction, hydrolysis, and other reaction types.
- *Nanozyme activity* Denotes the overall catalytic capability of nanomaterials with enzyme-like functions, typically encompassing factors such as the number of active sites, selectivity, and stability.
- *Biomimetic catalysis* Refers to strategies that achieve efficient catalytic reactions by mimicking the structure and mechanism of natural enzymes or biological catalytic systems.
- *Eco-nanozyme* Specifically refers to nanozymes applied in ecological contexts, such as energy conversion, pollutant degradation, or biogeochemical cycling.
- *Eco-nanozymology* A concept proposed in this review, emphasizing the application and study of nanozymes in energy, environmental, and ecosystem-level functions.

In this article, these terms follow a unified standard. The full term with abbreviation is used upon first mention, and subsequent references use the abbreviation to simplify the text.
